# Bioinspired supramolecular thermistor for resolving subtle temperature variations

**DOI:** 10.1126/sciadv.aei0002

**Published:** 2026-10-14

**Authors:** Yangtao Yu, Yahui Li, Dongyuan Zhang, Haoqi Han, Xinyu Ren, Kaiqi Gao, Haozhe Wen, Jiaxiang Wang, Zhiyuan Hu, Han Ren, Sicheng Chen, Ruiqing Ge, Yu Chang, Mingyi Chen, Hesheng Wang, Zhuoqing Yang, Yuanjin Zheng

**Affiliations:** ^1^ National Key Laboratory of Advanced Micro and Nano Manufacture Technology, Shanghai Jiao Tong University, Shanghai 200240, China.; ^2^ School of Integrated Circuits (School of Information Science and Electronic Engineering), Shanghai Jiao Tong University, Shanghai 200240, China.; ^3^ School of Electrical and Electronic Engineering, Nanyang Technological University, Singapore 639798, Singapore.; ^4^ School of Automation and Intelligent Sensing, Shanghai Jiao Tong University, Shanghai 200240, China.; ^5^ Institute for Digital Molecular Analytics and Science (IDMxS), Nanyang Technological University, Singapore 639798, Singapore.; ^6^ Mechanical Engineering College, Xi’an Jiaotong University, Xi’an 710049, China.; ^7^ Bionic Sensing and Intelligence Center (BSIC), Institute of Biomedical and Health Engineering, Shenzhen Institutes of Advanced Technology, Chinese Academy of Sciences, Shenzhen 518055, China.; ^8^ School of Biomedical Engineering, Division of Life Sciences and Medicine, University of Science and Technology of China, Hefei 230027, China.; ^9^ Suzhou Institute for Advanced Research, University of Science and Technology of China, Suzhou 215123, China.

## Abstract

Precise detection of subtle temperature variations under cryogenic conditions is critical for environmental monitoring but remains challenging because conventional thermistors suffer from limited sensitivity and mechanical interference. Here, we report a bioinspired supramolecular thermistor that achieves thermal transduction through molecularly gated charge transport. The hybrid polyurethane/carboxymethyl cellulose (PU/CMC)/MXene/NiO composite integrates dynamic hydrogen-bond reconfiguration, hydrated ionic conduction, and temperature-dependent electronic transport. Upon cooling, cooperative supramolecular evolution continuously modulates coupled ionic and electronic pathways, producing an ultrahigh negative temperature coefficient of −22,280% °C^−1^ between −80° and −75°C. The thermistor resolves detectable temperature changes below 0.01°C while maintaining negligible hysteresis, excellent reproducibility, and effective thermal-mechanical decoupling. Combined with a dedicated low-noise readout circuit, it enables reliable monitoring of subtle temperature evolution during simulated atmospheric cooling. Integrated into a multichannel sensor array, it further provides force-independent thermal mapping for robotic manipulation. This work establishes molecularly gated supramolecular thermal transduction as a strategy for ultrahigh-resolution cryogenic temperature sensing.

## INTRODUCTION

Thermal perception plays a fundamental role in how living systems interact with their surroundings, enabling organisms to detect environmental changes, maintain physiological homeostasis, and respond to potentially harmful stimuli (*1*–*5*). Translating such capabilities into artificial systems is increasingly important for emerging technologies, including soft robotics, wearable electronics, and environmental monitoring platforms operating in dynamic and uncertain environments (*6*–*11*). In these applications, accurately resolving subtle temperature fluctuations with high stability, low noise, and minimal mechanical interference remains a critical challenge. Conventional temperature sensors, including thermistors (*12*–*17*), conductive composites (*18*–*22*), and ionic gels (*23*–*26*), generally rely on temperature-dependent carrier transport within bulk materials, such as electronic conduction or ionic migration. Although these approaches have enabled diverse sensing platforms, achieving ultrahigh sensitivity while maintaining stable operation under extreme temperature conditions remains difficult.

The Biological thermal sensing systems provide an alternative paradigm for overcoming these limitations (*27*–*30*), where the thermoreceptors convert temperature stimuli into electrical signals through coordinated molecular processes. Temperature-induced structural reconfiguration regulates charge transport across specialized ion channels, ultimately generating electrical activity with remarkable sensitivity and reliability. Members of the transient receptor potential (TRP) ion channel family are representative examples of this thermal transduction strategy (text S1). More broadly, biological thermosensation relies on the coupling between molecular structural dynamics and charge transport, forming the basis of thermal transduction (*31*–*35*).

Inspired by this biological thermal transduction principle, supramolecular materials have recently emerged as promising platforms for temperature sensing because their dynamic intermolecular interactions can regulate charge transport pathways in response to external stimuli (*36*, *37*). In particular, reversible hydrogen-bond networks provide a molecular framework capable of continuously reorganizing with temperature, thereby coupling structural evolution with ionic and electronic transport. Nevertheless, most reported supramolecular temperature sensors operate near ambient temperatures and remain limited in resolving extremely small temperature variations under cryogenic conditions, where both charge transport and network stability become increasingly challenging.

Here, we report a bioinspired supramolecular thermistor that translates the principle of biological thermal transduction into a synthetic material system. The sensing material consists of a multiscale supramolecular network comprising nickel oxide (NiO) nanoparticles and MXene nanosheets embedded within a polyurethane (PU) and carboxymethyl cellulose (CMC) matrix. Temperature-dependent reconfiguration of the hydrogen-bond network dynamically modulates coupled ion-electron transport, resulting in highly amplified electrical responses to minute thermal perturbations. This bioinspired architecture produces an ultrahigh negative temperature coefficient of resistance (−22,280% °C^−1^) within the cryogenic temperature window from −80° to −75°C, enabling a detectable temperature change below 0.01°C, together with negligible hysteresis, drift-free operation, and effective thermal-mechanical decoupling. Beyond material-level performance, the thermistor was further integrated into functional systems to demonstrate its practical applicability. In a simulated atmospheric cooling profile, the sensor faithfully resolved subtle cryogenic temperature fluctuations, demonstrating its capability for high-resolution environmental monitoring. Integrated onto robotic fingertips, it further provided real-time thermal perception for adaptive object manipulation independent of gripping force. These demonstrations highlight the potential of the proposed thermistor for intelligent environmental monitoring and adaptive robotic systems.

## RESULTS

### Bioinspired thermal transduction through molecularly gated ion channels

To illustrate the conceptual relationship between biological thermal transduction and the proposed supramolecular sensing strategy, Fig. 1A compares the general thermal transduction process in biological thermoreceptors with the signal encoding mechanism realized in our supramolecular material system. In biological thermosensation, temperature stimuli are first detected by specialized thermoreceptors, where thermally induced conformational rearrangements regulate ion-channel gating and the associated ionic flux, ultimately generating electrical signals that encode thermal information. Inspired by this biological transduction principle, we implement an analogous sequence of thermally responsive processes within a supramolecular composite. Specifically, temperature-dependent reconfiguration of the hydrogen-bond network within the PU/CMC matrix dynamically modulates the local molecular configuration and transport pathways. The hydrated ionic environment within the PU/CMC matrix supports ionic transport, while progressive hydration of NiO at low temperatures promotes the formation of electrically insulating nickel hydroxides. Meanwhile, the strengthened hydrogen-bond network together with the reduced thermally activated electronic transport cooperatively suppresses both ionic and electronic conduction, resulting in pronounced resistance modulation under small temperature perturbations.

**Fig. 1.**
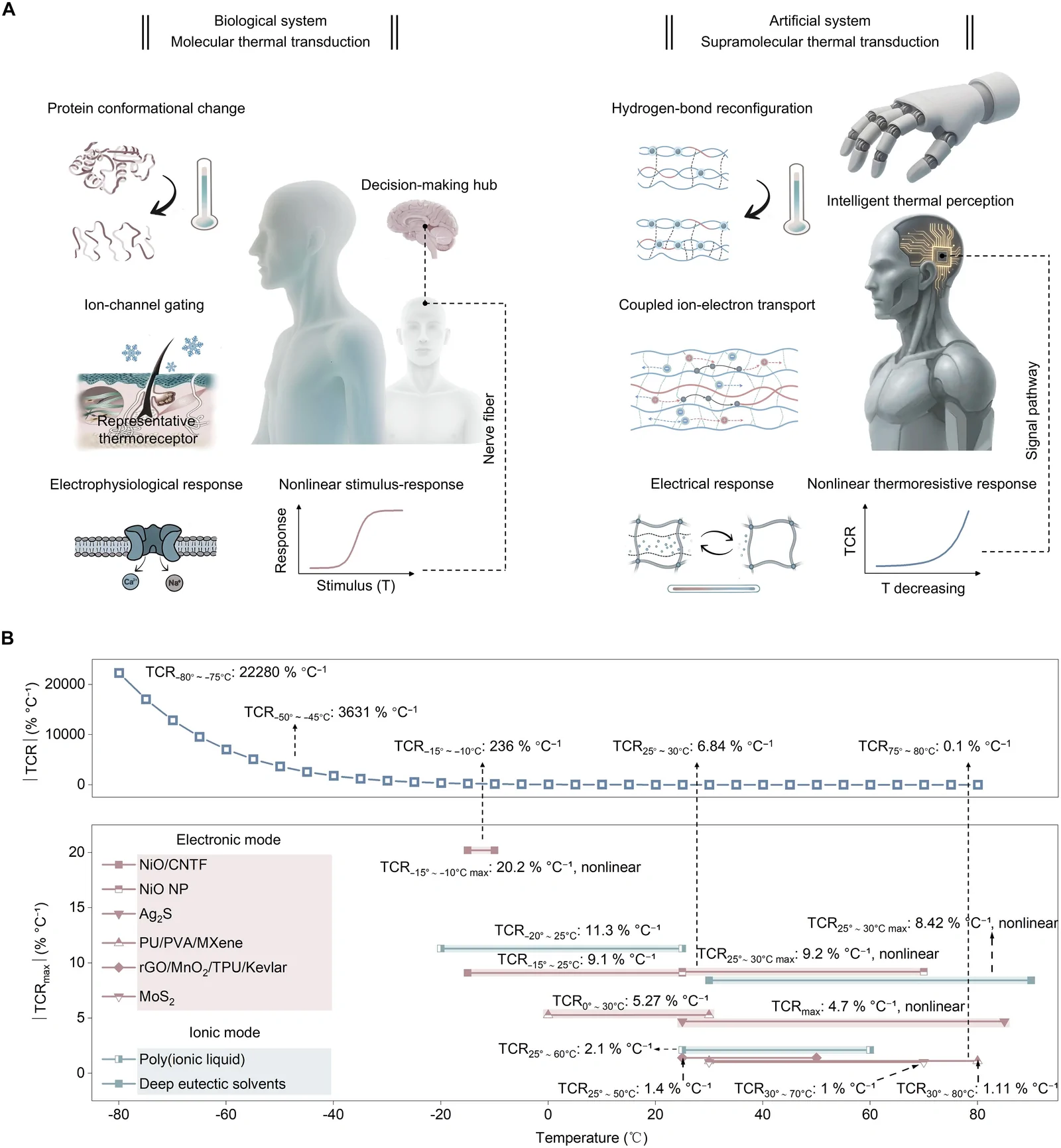
Bioinspired supramolecular thermal transduction and performance comparison. (**A**) Comparison between molecular thermal transduction in biological thermoreceptors and supramolecular thermal transduction in the proposed artificial system. (**B**) Temperature coefficient of resistance (TCR) as a function of temperature for the proposed sensor and representative thermal sensing materials reported in the literatures.

Figure 1B compares the performance of the proposed supramolecular thermistor with representative state-of-the-art thermal sensing materials, focusing on the temperature coefficient of resistance (TCR) and operational temperature range. Previously reported systems include multiphase composite sensors (*12*), semiconductor-based thermistors (*13*–*16*, *18*), and ionic hydrogel thermistors (*26*, *38*), which typically exhibit TCR values in the range of approximately 1–20% °C^−1^. By comparison, the bioinspired supramolecular thermistor developed here exhibits an ultrahigh TCR of 22,280% °C^−1^ within the cryogenic temperature window from −80° to −75°C (table S1). This exceptional thermoresistive response originates from the temperature-dependent reconfiguration of the supramolecular network, which dynamically regulates coupled ionic and electronic transport pathways responsible for thermal signal transduction. Notably, the TCR increases exponentially with decreasing temperature, while remaining approximately 1.25% °C^−1^ even at 45°C, demonstrating a broad operating range with progressively enhanced thermosensitivity toward cryogenic temperatures.

### Mechanistic framework of molecularly gated thermal transduction

We first examined the thermoresistive behavior of three representative systems: an electronic conduction network, an ionic conduction system, and the supramolecular composite (Fig. 2A). Across the temperature range from −80° to 80°C, the supramolecular system exhibits a markedly stronger negative temperature coefficient of resistance than the other two systems. While the resistance ratio (*R/R_0_*) of all three materials decreases at elevated temperatures, the supramolecular network maintains a more pronounced temperature dependence throughout the measurement range (inset in the Fig. 2A). The underlying charge transport processes in the individual components are summarized in Fig. 2B. In NiO, electronic conduction originates from thermally activated hole hopping associated with oxygen vacancy migration. In contrast, the CMC matrix supports ionic conduction governed by temperature-dependent ion mobility (text S2). Although both processes follow Arrhenius-type behavior, they differ in carrier species, transport pathways, and activation energies.

**Fig. 2.**
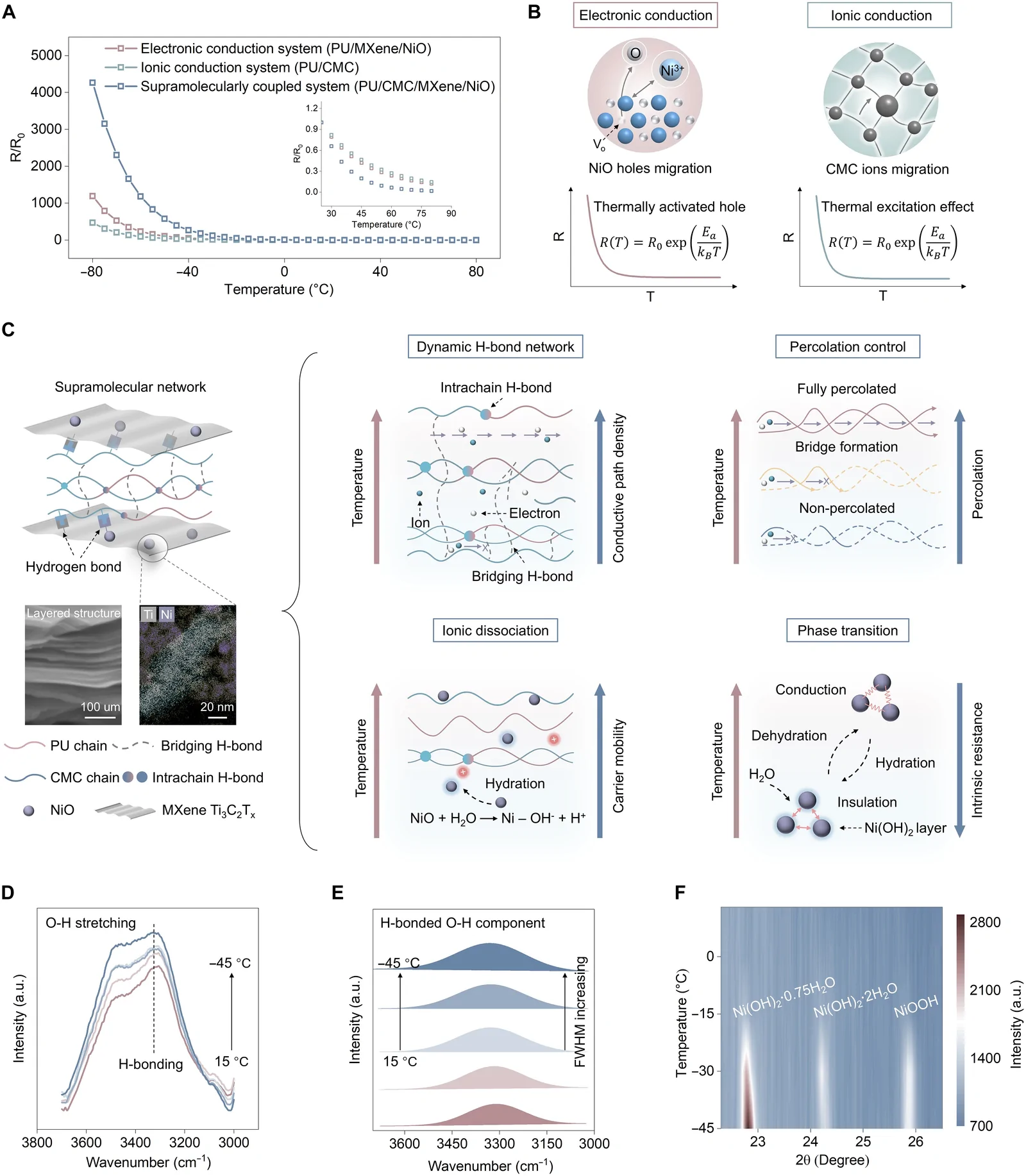
Supramolecularly coupled thermal transduction mechanism. (**A**) Temperature-dependent resistance response of electronic, ionic, and supramolecularly coupled material systems. (**B**) Schematic illustrations of the two primary charge-transport pathways: electronic conduction associated with hole transport in NiO, and ionic conduction arising from ion migration in the CMC matrix. (**C**) Proposed supramolecular transduction processes in the composite material, including hydrogen-bond network rearrangement, hydration-mediated ionic dissociation, modulation of percolative conduction pathways, and temperature-dependent phase transition. (**D** and **E**) In-situ FTIR spectra of O-H stretching modes and corresponding peak-width evolution, indicating changes in hydrogen-bond interactions. (**F**) In-situ x-ray diffraction mapping showing temperature-dependent structural evolution and hydration state transitions in the NiO-based component.

The supramolecular composite integrates these conduction channels within a multiscale architecture (Fig. 2C). A dynamic hydrogen-bond network connects the PU and CMC polymer chains with MXene nanosheets and dispersed NiO nanoparticles (fig. S1). These interactions provide structural cohesion while enabling temperature-responsive modulation. SEM reveals a layered morphology, suggesting anisotropic organization within the composite. Elemental mapping further confirms the nanoscale co-localization of Ti from MXene and Ni from NiO, indicating effective interfacial integration between the components. To understand the origin of the pronounced temperature coefficient of resistance in the supramolecular system, the conduction behavior can be interpreted as the result of several coupled structural and chemical processes. As the temperature decreases, strengthening of the supramolecular hydrogen-bond network reduces polymer chain mobility and alters the organization of conductive microdomains within the composite. These structural changes are expected to influence the connectivity of the conductive pathways formed by MXene sheets and NiO nanoparticles. Consequently, the conductive network may approach a partially percolated state, which restricts both ionic and electronic transport and results in an increase in resistance. At the same time, cooling promotes hydration processes at the surface of the dispersed NiO nanoparticles. Adsorbed water molecules facilitate partial ionic dissociation, generating mobile species such as H^+^ and OH^−^ that locally modify the carrier environment. With continued hydration, the NiO surface undergoes a progressive chemical transformation, during which hydroxide species accumulate and promote the formation of Ni(OH)_2_ and hydrated intermediate layers. These hydroxide phases form electrically insulating shells around the NiO cores, interrupting long-range conductive pathways within the composite. The combined effects of supramolecular network restructuring, hydration-driven ionic processes, and the formation of insulating hydroxide layers co-contribute to the nonlinear resistance increase observed at low temperatures.

To validate these coupled effects, Fourier transform infrared (FTIR) spectroscopy (Fig. 2D and fig. S2A) reveals a pronounced blue shift and increased intensity of the O-H stretching band as temperature decreases, consistent with strengthened hydrogen bonding (*39*). This is confirmed by a marked broadening in full width at half maximum (FWHM) (Fig. 2E, fig. S2B), indicating denser supramolecular interactions at lower temperatures. Consistent temperature-dependent blue shifting is also observed for other hydrogen-bond-related vibrations, such as the C=O stretching band (fig. S2, C and D), further supporting the strengthening and reorganization of the supramolecular hydrogen-bond network at lower temperatures. Simultaneously, in-situ x-ray diffraction (XRD) mapping (Fig. 2F, figs. S3 and S4) confirms a progressive phase evolution from crystalline NiO to hydrated Ni(OH)_2_ species (*40*, *41*). The selected area electron diffraction (SAED) patterns (fig. S5 and text S3) illustrate a temperature-induced phase transition, where the initially ordered crystalline structure at room temperature transforms into an amorphous phase at low temperatures. This hydration-induced structural transition disrupts conduction continuity, thereby inducing a substantial resistance jump. Electrochemical impedance spectroscopy (EIS) measurements further support the proposed transport mechanism (fig. S6). Both the frequency-dependent impedance spectra and Nyquist plots show an increased resistive response upon cooling, indicating that charge transport becomes hindered at lower temperatures. Equivalent-circuit fitting further reveals concurrent increases in both the ionic and electronic transport resistances. The increase in ionic resistance is attributed to the reduced mobility of ions within the PU/CMC supramolecular network as hydrogen-bond reconfiguration suppresses ion migration. Meanwhile, the electronic resistance increases because lower temperatures weaken thermally activated electron transport and disrupt the electronic percolation pathways through the temperature-induced formation of nickel hydroxide species.

### Thermal-mechanical decoupling and robustness

Reliable temperature sensing requires minimal interference from external mechanical deformation, especially in flexible devices and robotic systems where temperature and pressure often coexist. To evaluate the thermal-mechanical decoupling capability of the supramolecular thermistor, we measured its resistance response under sequential pressure loading from 2 kPa to 1 MPa at different temperatures. As shown in Fig. 3A, the hydrogen-bonded supramolecular thermistor maintains a nearly stable normalized resistance under repeated pressure loading at 25°C, 0°C, and −20°C, indicating that external pressure has negligible influence on its thermal readout over a broad temperature range. In contrast, the non-hydrogen-bonded control shows obvious resistance drops under the same pressure-loading conditions (Fig. 3B), suggesting that its conductive structure is readily disturbed by mechanical deformation. The pressure sensitivity was further quantified in Fig. 3C. The hydrogen-bonded system shows nearly zero pressure sensitivity across 0–1000 kPa, whereas the control exhibits a pronounced pressure-dependent resistance decrease, with a maximum sensitivity of 0.09 kPa^−1^. These results confirm that the hydrogen-bonded supramolecular network effectively suppresses pressure-induced electrical interference and preserves stable thermal sensing.

**Fig. 3.**
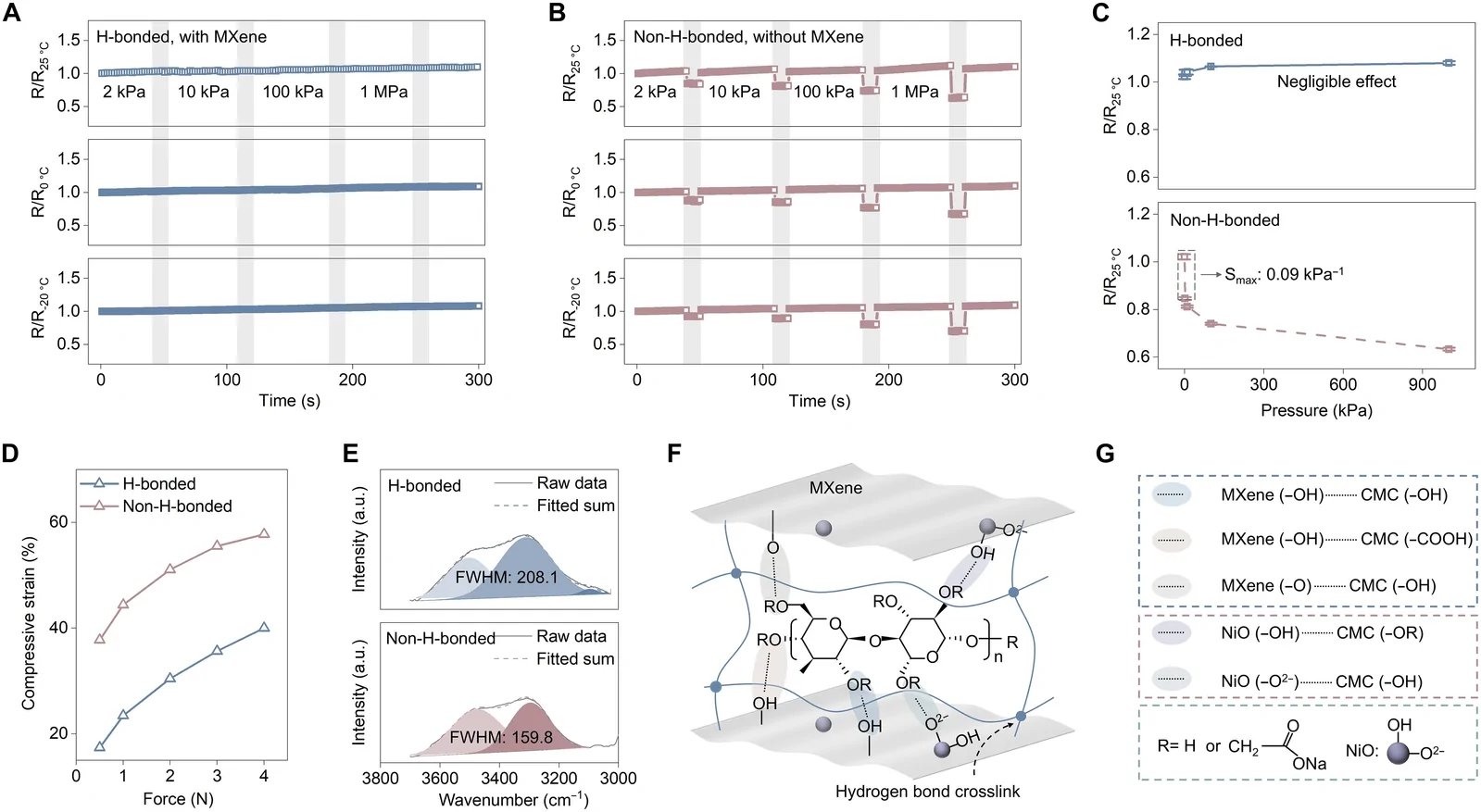
Thermal-mechanical decoupling enabled by supramolecular hydrogen-bond network. (**A**) Pressure response of the hydrogen-bonded supramolecular thermistor at different temperatures. (**B**) Pressure response of the non-hydrogen-bonded control at different temperatures. (**C**) Comparison of pressure sensitivity between hydrogen-bonded and non-hydrogen-bonded systems. (**D**) Comparison of compressive mechanical behavior of hydrogen-bonded and non-hydrogen-bonded composite. (**E**) Deconvolution of the hydrogen-bonded O-H stretching band and broadening of the hydrogen-bond component. (**F**) Schematic illustration of hydrogen-bond crosslinking between CMC chains, MXene nanosheets and NiO particles. (**G**) Representative hydrogen-bond configurations formed among CMC, MXene, and NiO.

Mechanical compression tests (Fig. 3D) show that the hydrogen-bonded composite sustains larger compressive strain than the non-hydrogen-bonded control under identical loading, indicating improved mechanical robustness (fig. S7). The enhanced mechanical robustness arises from a dynamic supramolecular network formed via hydrogen bonding among CMC chains, NiO nanoparticles, and MXene nanosheets. FTIR analysis further supports the presence of stronger intermolecular interactions in the hydrogen-bonded system. The deconvoluted hydrogen-bonded O-H component exhibits a larger FWHM than that of the control (Fig. 3E), while the C=O stretching band shows a distinct blue shift (fig. S8), together indicating strengthened hydrogen bonding and enhanced supramolecular interactions. Hydrogen bonds are established between the hydroxyl, carboxyl, and alkoxy groups of CMC and the oxygen-containing surface groups of MXene and NiO, forming an interconnected supramolecular network (Fig. 3, F and G). Among these interactions, the CMC-MXene interface plays the dominant role in mechanical stabilization. The flexible CMC chains bridge adjacent MXene nanosheets through multiple hydrogen bonds, generating continuous interfacial crosslinks across the conductive framework (fig. S9). This interconnected architecture effectively suppresses nanosheet slippage and preserves structural integrity during compression. To further clarify the respective contributions of MXene and NiO, additional control experiments were performed (fig. S10). Although hydrogen bonding between CMC and NiO also improves the mechanical stability of the composite, its reinforcement is noticeably weaker than that provided by the CMC-MXene network. This result indicates that NiO mainly serves as a dispersed conductive phase, whereas MXene functions as the primary structural scaffold that stabilizes the supramolecular network. The resulting stabilization mechanism is illustrated in fig. S11. In the hydrogen-bonded system, the CMC-MXene network maintains conductive pathway continuity during external compression by preserving stable interfacial contacts, resulting in minimal variation in interfacial contact resistance and nearly constant overall resistance. In contrast, without these hydrogen-bond bridges, local structural collapse readily disrupts the conductive pathways, leading to pronounced resistance changes under mechanical loading.

### Thermal sensing performance and detection of subtle temperature variations

The normalized resistance (*R/R_0_*) of the sensor was measured over a wide temperature range from −60° to 80°C under programmed thermal cycling by an independent third-party testing laboratory (Fig. 4A). As the temperature decreases into the sub-zero regime, the resistance increases markedly, reaching a maximum R/R_0_ value of approximately 1180 at −60°C. When the temperature returns to ambient (22°C) or higher levels (55° and 80°C), the resistance rapidly decreases and stabilizes at values below 1.3, indicating a reversible thermoresistive response of the material system (fig. S12). The inset highlights the resistance variation at temperatures below room temperature, where the sensor resolves temperature changes on the order of ∼0.01°C^−1^. The thermal sensor also demonstrates stable operation under constant temperature conditions, the normalized resistance remains nearly unchanged, indicating negligible thermal drift (Fig. 4B and fig. S13, A and B). The thermal hysteresis behavior was evaluated at three representative cryogenic temperatures (−10°C, −50°C, and −90°C) (Fig. 4C, fig. S13, C and D, and text S4). Only minor hysteresis is observed during repeated heating-cooling cycles, indicating reversible thermoresistive response under deep-cooling conditions (Fig. 4D). To further analyze the temperature dependence of the resistance response, the *R/R_0_*-*T* relationship was fitted using an exponential model (Fig. 4E). The fitting reveals three distinct temperature regimes characterized by different transport behaviors. At elevated temperatures (20° to 80°C) (fig. S14A), the resistance exhibits a gradual exponential decay, consistent with thermally activated carrier transport. In the intermediate temperature range (−30° to 20°C) (fig. S14B), the slope becomes steeper, suggesting stronger coupling between ionic motion and the hydrogen-bonded supramolecular network. In the lower temperature regime (−80° to −30°C), the resistance changes more sharply with temperature, indicating enhanced sensitivity of the transport pathways to structural variations within the supramolecular network. Figure S15 compares the experimental *R/R_0_* values with those calculated from the fitted model over the full temperature range from −80° to 80°C. The close agreement between the measured data and fitted results confirms the validity of the fitting approach. To further evaluate fabrication reproducibility, sensors fabricated from different batches exhibit nearly identical temperature-dependent responses over the entire measurement range (fig. S16A). In addition, sensors fabricated within the same batch show a relative standard deviation below 2% (fig. S16B), indicating excellent sample-to-sample uniformity. Furthermore, varying the sensing film thickness from 0.75 μm to 0.90 μm produces similar thermal responses (fig. S16C), demonstrating that the sensing behavior is robust against small variations in film thickness.

**Fig. 4.**
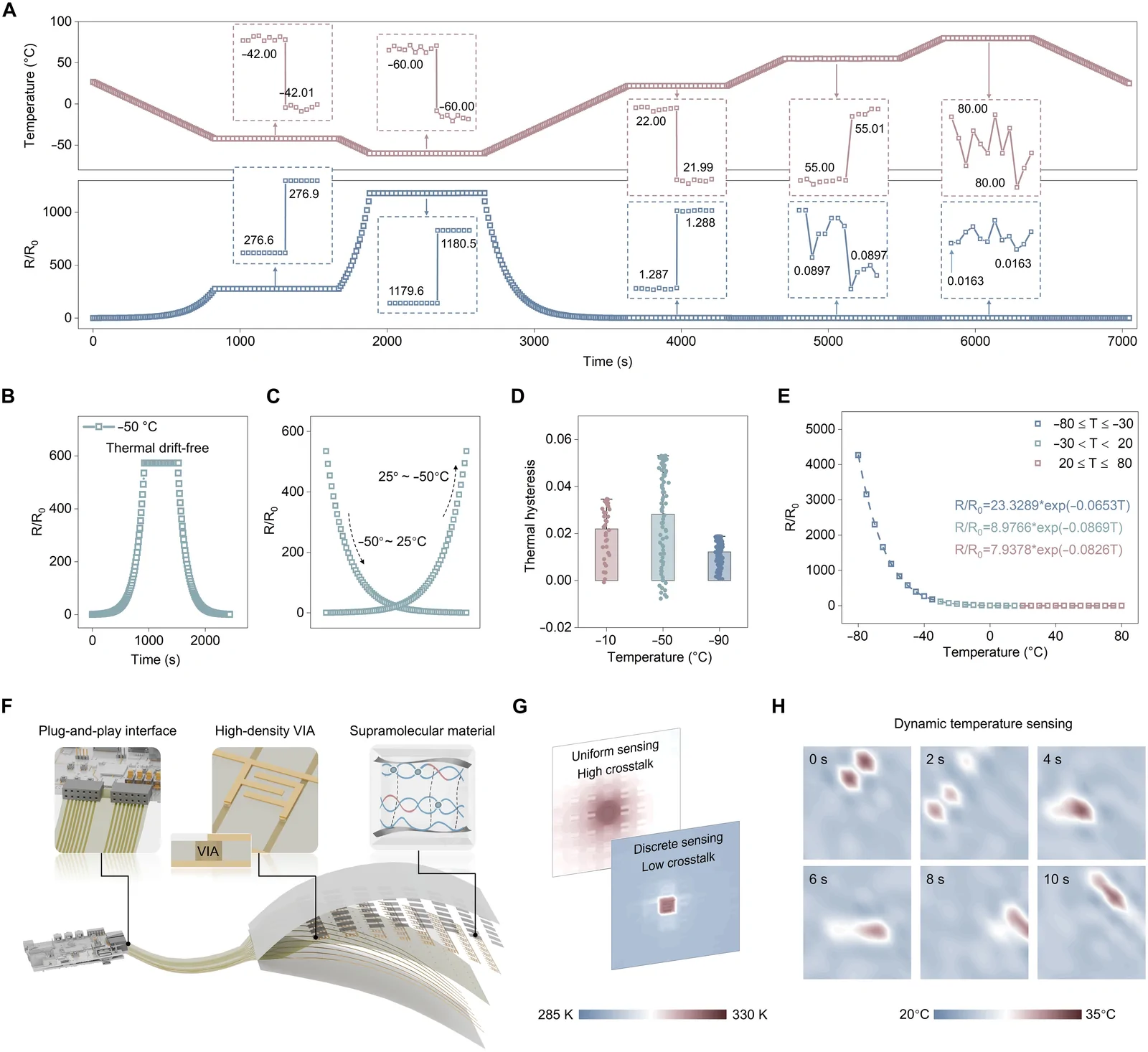
Thermal sensing with ultrahigh resolution and scalable array integration for temperature mapping. (**A**) Real-time monitoring of normalized resistance and temperature during thermal loading (−60° to 80°C). The system demonstrates a detectable temperature change below 0.01°C, enabling precise discrimination of minute temperature fluctuations. Insets highlight the reproducible stepwise transitions and the reliable thermal responsiveness. (**B**) Thermal drift-free sensing performance during prolonged operation at −50°C. (**C**) Reversible resistance response during repeated temperature cycling between 25° and −50°C. (**D**) Quantitative thermal hysteresis analysis under different thermal zones (−10°C, −50°C, and −90°C), revealing minimal hysteresis. (**E**) Exponential fitting of the *R/R_0_*-*T* relationship across three thermal regimes, demonstrating nonlinearity and regime-specific response kinetics. (**F**) Flexible multichannel sensing system with plug-and-play interconnection, high-density VIAs, and the supramolecular sensor array. (**G**) Thermal simulations of continuous and discrete sensing architectures with corresponding temperature distributions. (**H**) Time-resolved thermal mapping of a moving heat source across the sensor array.

The integrated sensor architecture combines a supramolecular sensing film with high-density vertical interconnect access (VIA) structures and a plug-and-play electrical interface (Fig. 4F). The supramolecular layer serves as the temperature-responsive element, while the VIA structures provide vertical electrical connections that enable multilayer signal routing. The standardized interface further allows modular integration with external signal acquisition modules, facilitating system-level assemble (figs. S17 and S18). To examine the influence of sensing architecture on thermal cross-talk, comparative thermal simulations were performed for two representative designs: a uniform sensing layout and a discrete sensing configuration (Fig. 4G and fig. S19). In the uniform architecture, thermal stimulation produces a relatively broad response region, leading to noticeable signal spreading across neighboring sensing units. By contrast, the discrete sensing configuration confines the thermal response within localized regions, thereby reducing signal overlap between adjacent sensing units. The spatial sensing capability of the array was further evaluated through dynamic trajectory mapping experiments (fig. S20). As the thermal stimulus moves across the array surface, the sensor output generates a continuous spatiotemporal signal pattern corresponding to the motion trajectory. Real-time thermal mapping (Fig. 4H) demonstrates that the array can track localized temperature variations over time, producing a clear thermal response path (fig. S21). In addition to dynamic tracking, the sensor array can also resolve static temperature distributions across the sensing surface (fig. S22). The resulting thermal maps show well-defined spatial patterns with limited signal spreading, indicating effective spatial discrimination of temperature signals.

### Ultrahigh-resolution environmental sensing and robotic thermal perception

To demonstrate the practical relevance of the proposed supramolecular thermistor, we selected two representative application scenarios that directly evaluate its two key capabilities: ultrahigh-resolution temperature sensing under cryogenic conditions and reliable thermal perception under simultaneous mechanical interaction. The first application targets atmospheric temperature monitoring. Precise measurement of subtle temperature variations in the upper atmosphere is essential for atmospheric research, climate studies, and environmental monitoring, where temperatures commonly decrease below −60°C during radiosonde ascent. Although radiosondes are equipped with commercial temperature sensors, achieving high-resolution detection under cryogenic conditions remains challenging because sensor sensitivity generally decreases as temperature falls. Rather than replacing existing radiosonde instrumentation, our objective is to demonstrate the capability of the proposed thermistor to resolve minute temperature variations throughout a representative atmospheric cooling process. To achieve this in a controllable laboratory environment, we reproduced the temperature trajectory encountered during balloon ascent using a physical property measurement system (PPMS), which enables programmable thermal cycling between room temperature and −60°C (Fig. 5A). A major challenge in this measurement arises from the intrinsically high resistance of the supramolecular sensing material. While the baseline resistance reaches hundreds of megaohms, the resistance variation associated with a temperature change of 0.01°C is only several tens of ohms. Accurate readout therefore requires both ultralow electrical noise and high measurement resolution. To address this challenge, we developed a dedicated low-noise acquisition circuit (Fig. 5B), consisting of a switchable precision reference resistor network, an ultra-low-noise low-dropout regulator, a high-input-impedance buffer, and a 24-bit delta-sigma analog-to-digital converter (ADS1247). Digital oversampling and averaging implemented by the microcontroller further suppress stochastic noise and improve the effective resolution. Using this system, the sensor continuously tracked the simulated atmospheric cooling profile (fig. S23 and text S5). As shown in Fig. 5C, the thermistor exhibits a stable resistance response throughout the entire cooling process down to −60°C and reliably resolves temperature variations as small as 0.01°C, demonstrating its capability for monitoring subtle thermal fluctuations under representative cryogenic environmental conditions.

**Fig. 5.**
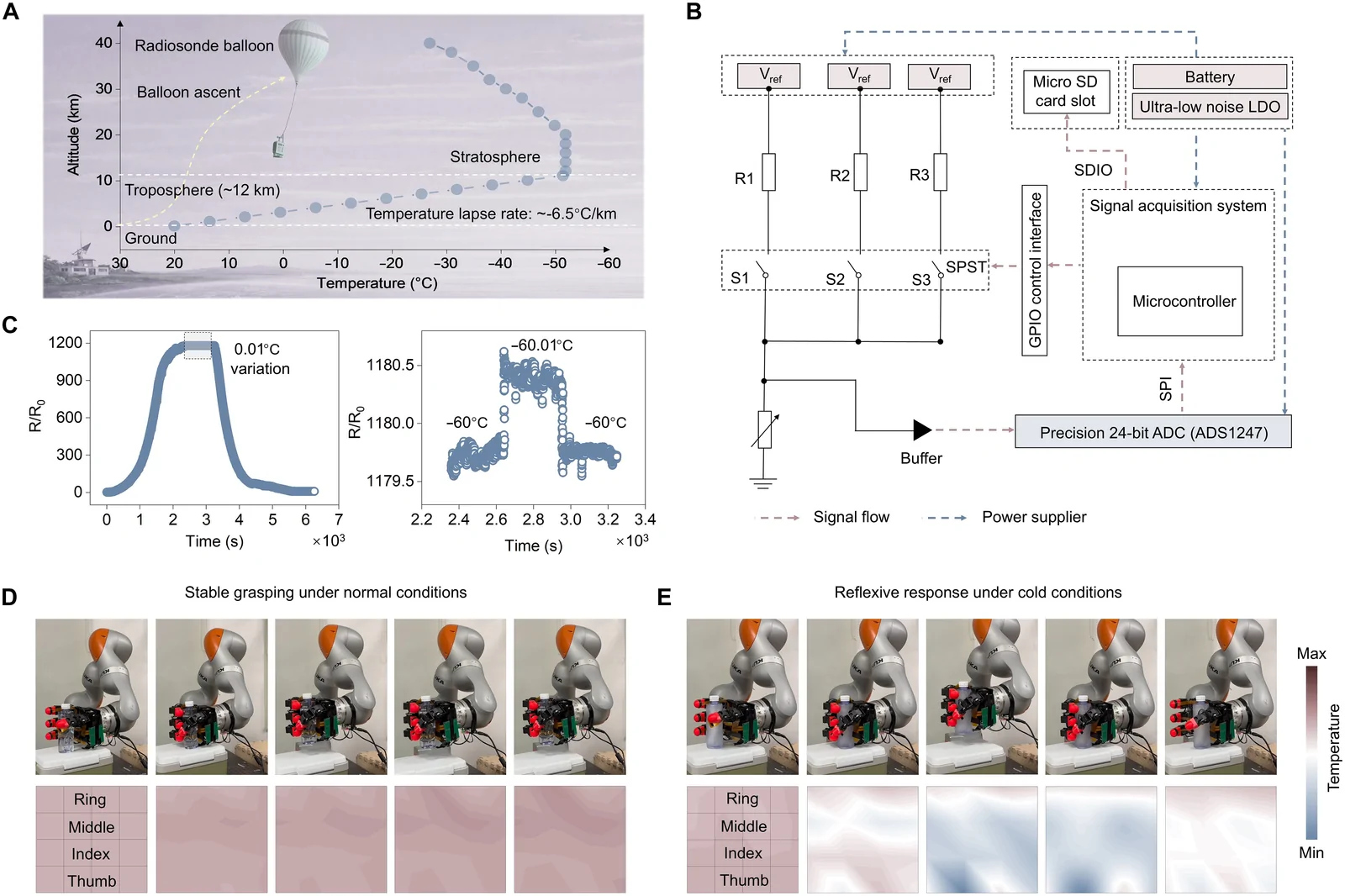
Ultrahigh-resolution environmental sensing and robotic thermal perception. (**A**) Simulated atmospheric temperature profile during radiosonde balloon ascent, showing the temperature decrease in the troposphere toward the tropopause (∼12 km, ∼−60°C). (**B**) Architecture of the high-precision signal acquisition system designed for ultrahigh-resistance thermistor readout. (**C**) Representative resistance response of the supramolecular thermistor during simulated atmospheric cooling. The sensor resolves temperature variations as small as 0.01°C, demonstrating ultrahigh sensitivity in the cryogenic regime. (**D**) Robotic manipulation under normal conditions with stable temperature mapping on the fingertips. (**E**) Thermal response under cold stimuli, where the robotic hand detects temperature changes and triggers adaptive release. Colors indicate the temperature distribution across the sensing channels.

The second application focuses on intelligent robotic thermal perception, where accurate temperature sensing should be reliable during physical contact with objects. Unlike the atmospheric demonstration, which evaluates sensing performance under extreme thermal conditions, this application examines whether the thermistor can preserve accurate thermal perception in the presence of mechanical loading. To this end, a 16-channel sensor array was fabricated and integrated onto robotic fingertips together with the signal acquisition and control electronics (figs. S24 and S25). Under normal operating conditions, the robotic hand maintains stable grasping behavior (Fig. 5D and movie S1). Upon contacting a cold object, the sensor array rapidly identifies the local temperature distribution and triggers an autonomous release response through closed-loop control (Fig. 5E and movie S2). Increasing the grasping force produces negligible changes in the measured temperature distribution (fig. S26), confirming that thermal perception remains stable under varying mechanical loads. These results demonstrate that the proposed supramolecular thermistor combines ultrahigh thermal sensitivity with effective thermal-mechanical decoupling, enabling reliable operation in both cryogenic environmental monitoring and intelligent robotic systems.

## DISCUSSION

In summary, we developed a bioinspired supramolecular thermistor that implements a molecularly gated thermal transduction strategy for ultrahigh-resolution temperature sensing under cryogenic conditions. The hybrid PU/CMC/MXene/NiO composite integrates dynamic hydrogen-bond evolution, hydrated ionic conduction, and temperature-dependent electronic transport into a coupled supramolecular network, enabling continuous modulation of charge transport during cooling. This cooperative molecular response produces an ultrahigh negative temperature coefficient, resolves detectable temperature changes below 0.01°C, and simultaneously provides negligible hysteresis, excellent reproducibility, and effective thermal-mechanical decoupling. Combined with a dedicated low-noise electronic readout, the thermistor continuously tracks subtle temperature evolution during simulated atmospheric cooling and further enables force-independent thermal perception and adaptive feedback when configured as a multichannel robotic sensor array. Beyond the performance demonstrated here, this work demonstrates that dynamic supramolecular interactions can serve not only as structural motifs but also as active molecular gates for regulating thermal transduction. Rather than relying solely on the intrinsic properties of conventional semiconductors, the sensing behavior is governed by cooperative evolution of hydrogen bonding, ionic transport, and electronic conduction, providing a complementary route for constructing highly nonlinear thermoresistive responses. We anticipate that this molecularly gated thermal transduction strategy will provide a framework for developing thermal interfaces for cryogenic environmental monitoring, intelligent robotics, wearable electronics, and other adaptive sensing technologies.

## MATERIALS AND METHODS

### Materials

Water-borne polyurethane (PU) was sourced from Shenzhen Jitian Chemical Co., Ltd. (China). Two-dimensional transition metal carbide MXene (Ti_3_C_2_T_x_, 400 mesh) was obtained from FoShan Xinxi Technology Co., Ltd. (China). Carboxymethyl cellulose (CMC) was purchased from Sinopharm Chemical Reagent Co., Ltd. (China). A commercially available dispersion of nickel oxide (NiO) was purchased from Dashinou Nanotechnology (Changzhou) Co., Ltd. (China). Ultrapure deionized (DI) water with a resistivity of 18.2 megaohm·cm was used throughout all experiments. All reagents were used as received without further purification.

### Design rationale of the sensing composite

The sensing composite was designed by integrating four functional components with complementary roles to achieve coupled supramolecular thermal transduction. PU serves as the flexible polymer matrix that provides the structural framework of the composite. CMC introduces abundant hydroxyl and carboxyl groups, enabling the formation of a dynamic hydrogen-bond network while simultaneously providing hydrated ionic conduction pathways. NiO nanoparticles function as the primary electronic conductive phase, where temperature-dependent carrier transport contributes to the thermoresistive response (fig. S27A). MXene nanosheets are incorporated primarily as an interfacial reinforcing component rather than a thermosensitive component. Although MXene itself exhibits a positive temperature coefficient (PTC) behavior (fig. S27B), whereas the composite displays a pronounced negative temperature coefficient (NTC), its incorporation improves the structural stability of the supramolecular network through extensive hydrogen-bond interactions with CMC. Comparative experiments further show that the MXene-free composite exhibits an even larger TCR but suffers from pronounced pressure-induced signal fluctuations, indicating that the primary role of MXene is to stabilize conductive pathways and suppress mechanically induced microstructural collapse rather than directly enhance temperature sensitivity.

### Synthesis of the thermosensitive film

The thermoresistive composite film was prepared via a precision-controlled slit-coating strategy. Initially, 7.38 g of water-borne PU and 0.12 g of CMC were co-dissolved in DI water under magnetic agitation (1500 rpm) at ambient temperature for 2 h, yielding a stable polymeric matrix. Subsequently, 0.5 g of Ti_3_C_2_T_x_ MXene nanosheets was incorporated into the matrix, followed by ultrasonication in an ice-cooled bath (30 min) to facilitate nanoscale dispersion and prevent restacking. A measured quantity of NiO dispersion (2.0 g) was then introduced into the pre-hybridized solution, which was further homogenized via continuous magnetic stirring (10 h, 1500 rpm) to ensure colloidal stability and uniformity. The resulting nanocomposite ink was subjected to vacuum degassing (30 min) to eliminate entrapped air, and subsequently cast onto pre-cleaned polyethylene terephthalate (PET) substrates using a calibrated slit die coater. Final thermal curing was conducted at 80°C for 3 h under ambient atmosphere, yielding a dense and uniform sensing layer with a target thickness of ∼200 μm.

### Fabrication of high-density interconnected thermal sensor array

The electrode array was fabricated using a commercially available flexible printed circuit board (FPCB), chosen for its technological maturity, structural scalability, and plug-and-play interface compatibility. A copper-polyimide-copper (Cu-PI-Cu) architecture was adopted in combination with via interconnects to realize the customized electrode layout. The electrodes were designed in an interdigitated configuration and vertically interconnected through vias, enabling spatial overlap of device layers and thereby enhancing the integration density of the sensor array. Compared to conventional sandwich-structured designs, our configuration employs only two functional layers, effectively reducing both the device thickness and fabrication complexity.

The as-fabricated thermoresistive composite film was placed onto a home-made Ecoflex elastomer substrate to facilitate subsequent patterning. Laser ablation (λ = 355 nm, pulse energy = 10 μJ, spot size ≈ 30 μm) was employed to define a high-resolution 8 by 8 pixel array, with each unit cell precisely structured into 2.0 by 2.0 mm^2^ squares. To encapsulate the patterned film, a thermoplastic ethylene-vinyl acetate (EVA) sheet was used to transfer the sensing array and thermally annealed at 80°C for 15 s, enhancing interfacial adhesion via localized melting and bonding. Following detachment of the Ecoflex support, the individual sensing elements were carefully aligned and transferred onto a pre-fabricated electrode array. Final integration was achieved through hot-pressing at 80°C under a compressive load of 2.5 MPa for 1 h, ensuring conformal contact and robust interfacial coupling between the sensing layer and the electrode network.

### In-situ XRD and FTIR analysis

To survey the physical phase composition, chemical structure, and crystallographic characteristics of the thermosensitive film, as well as to investigate the temperature-dependent crystallinity evolution, in situ XRD analysis was performed using an ARL EQUINOX 3500 diffractometer. Temperature-resolved diffraction patterns were acquired over a 2θ range of 2° to 90°, with a step size of 0.02° and a scan rate of 1°/min, under a controlled thermal environment spanning from −45° to 15°C. Real-time monitoring of phase transitions and lattice stability was achieved by tracking the temperature-induced shifts and intensity variations of characteristic diffraction peaks. Notably, the presence and intercalation behavior of CMC within the interlayer MXene nanosheets were probed via peak displacement in the low-angle region (∼5° to 7°), indicative of expanded interlayer spacing. Crystallographic changes at different thermal states were further analyzed by correlating experimental patterns with standard reference databases, enabling quantitative assessment of temperature-dependent crystallinity variations. In-situ FTIR spectroscopy was conducted using a Thermo Fisher Scientific Nicolet iS50 spectrometer equipped with a temperature-controlled sample stage to probe the thermally modulated supramolecular interactions within the composites. Spectra were recorded over a wavenumber range of 600–4000 cm^−1^ under a controlled temperature environment spanning −45° to 15°C. This temperature-resolved FTIR analysis was specifically designed to demonstrate the dynamic evolution of intermolecular bonding states, particularly the strength and abundance of hydrogen bonds-within the CMC-assisted supramolecular framework. Variations in the vibrational signatures corresponding to C=O, O-H, and C-O stretching modes were systematically monitored to track hydrogen bonding dynamics and molecular configuration under thermal stimuli. The formation or disruption of hydrogen bonds manifests as distinct changes in peak positions, intensities, and bandwidths. Quantitative insights into hydrogen bond strength and were extracted via fitting the FWHM of relevant peaks across the temperature range. These observations provide direct evidence of the thermally responsive supramolecular network modulation, which underlies the dynamic temperature sensing behavior reported in this work.

### Thermal response measurement

Accurate temperature calibration was carried out using a precision K-type thermocouple placed in close proximity to the sensing unit to eliminate spatial thermal lag. Calibration curves were established by correlating the normalized resistance change (*R/R_0_*) with reference temperatures incrementally varied in 5°C steps during a controlled thermal sweep from −80° to 80°C. Each calibration cycle was repeated three times to ensure measurement repeatability and thermal tracking accuracy.

To evaluate the temperature sensing resolution, measurements were performed on a Quantum Design PPMS-9 platform with temperature control precision down to 0.01°C (calibration was performed by a third-party testing institution). A stepwise increase of 0.01°C was applied to examine the film’s minimum detectable thermal response (fig. S28). The sensing element was electrically connected using the low-noise Vanderbilt wiring method, and the system was thermally and electromagnetically isolated using a sealed chamber with closed-loop feedback regulation. The noise floor and thermal resolution were quantitatively assessed by Allan deviation analysis, confirming the ability of sensor to detect sub-0.01°C temperature changes with high stability. The stability of the thermal sensor was initially evaluated by maintaining it at −10°C, −50°C, and −90°C for a period of time under isothermal conditions while continuously recording the resistance signal. Signal drift behavior was analyzed via statistically evaluating the *R/R_0_* values under isothermal conditions. Thermal hysteresis behavior was examined by comparing the *R/R_0_* values during independent heating and cooling cycles, performed with a step size of 0.5 K and a thermal ramp rate of 5 K/min. Hysteresis width and thermal reversibility were quantified by analyzing the deviation between heating and cooling curves at identical temperature points. The results demonstrate minimal hysteresis and excellent reversibility across multiple cycles, underscoring the supramolecular network’s thermodynamic stability and reversibility.

### High-precision wide-range signal acquisition circuit

To accurately resolve minute resistance variations while accommodating the several orders of magnitude resistance change of the supramolecular thermistor during cryogenic measurements, a wide dynamic range signal acquisition circuit was developed. Instead of using a single fixed reference resistor, the readout circuit employs multiple precision reference resistors combined with analog switches to realize automatic range switching. Three reference resistors corresponding to low, medium, and high resistance ranges are selectively connected by GPIO-controlled analog switches, only one reference resistor is active during each measurement. The selected reference resistor and the sensor resistance form a voltage divider, and the divider voltage is digitized by a 24-bit delta-sigma analog-to-digital converter (ADS1247), from which the sensor resistance is calculated.

The multi-range architecture maximizes the effective utilization of the ADC input range. When a single reference resistor is used, the divider output approaches either the supply voltage or ground if the sensor resistance differs substantially from the reference resistance, leading to poor voltage sensitivity and reduced resistance resolution. Therefore, the reference resistor is selected so that its resistance remains comparable to the sensor resistance, maintaining the divider voltage within approximately 20 to 80% of the ADC full-scale input range over the entire measurement process. During acquisition, the microcontroller first performs a coarse resistance estimation, automatically selects the appropriate measurement range, and switches to adjacent ranges whenever the measured voltage approaches the upper or lower threshold. After each range transition, sufficient settling time is allowed for the analog switch, buffer amplifier, and digital filter before valid data acquisition resumes.

To ensure accurate measurement of high-resistance states, the voltage divider is followed by a high-input impedance buffer amplifier, preventing ADC sampling from loading the sensor and minimizing leakage-induced measurement errors. The analog front end is powered by an ultra-low-noise low-dropout regulator (LDO), while distributed decoupling capacitors suppress power supply ripple and high frequency interference. Signal digitization is performed using the ADS1247 operating at a low data rate with its built-in sinc digital filter, and additional oversampling and digital averaging are implemented in the microcontroller to further improve the effective number of bits and suppress random noise. Furthermore, each measurement range is individually calibrated using low temperature coefficient precision reference resistors to compensate for gain and offset errors, while switch on-resistance, leakage current, input bias current, and PCB leakage are incorporated into the high-resistance calibration procedure.

### Simulated atmospheric temperature measurement

To evaluate the system-level temperature sensing capability under extreme conditions, a simulated atmospheric cooling experiment was performed to emulate the temperature evolution during radiosonde balloon ascent. The temperature program started from room temperature (20°C) and was decreased at a rate of 2°C min^−1^ for 40 min until −60°C was reached. The temperature was then maintained for 5 min, followed by a further decrease to −60.01°C and stabilization for another 5 min. Subsequently, the temperature was increased back to −60°C and held for 5 min before ramping up to 0°C at a rate of 1.5°C min^−1^, with a final stabilization period of 10 min. Temperature control was provided by a Quantum Design PPMS-9 T (EC-II) platform, which served solely as a programmable thermal environment. The packaged sensor was mounted onto the sample rod and inserted into the PPMS chamber. Electrical signals were not measured by the PPMS system, instead, resistance variations were recorded using the custom-designed high-precision signal acquisition circuit described above. This configuration enabled system-level evaluation of the sensor and readout electronics under precisely controlled thermal conditions.

### Communication protocol between the Bluetooth module and the host computer (robotic platform)

The communication between the Bluetooth module and the host computer (robotic platform) is established via a serial port protocol. The microcontroller unit (MCU) continuously transmits real-time data frames through a UART interface, which are wirelessly forwarded to the host computer via the Bluetooth module. On the host side, a Python script opens the designated serial port and continuously monitors incoming data in a high-frequency loop to ensure low-latency processing. Each valid data frame consists of 34 bytes, beginning with a start byte 0xFA and ending with a stop byte 0xAA. Incoming bytes are temporarily stored in a buffer, where the program scans for complete and valid frames based on these predefined markers. Once a valid frame is identified, it is immediately timestamped and logged to a CSV file for future analysis. Simultaneously, the frame is parsed into 16 16-bit analog-to-digital conversion (ADC) values.

The extracted ADC values are first converted to voltages using the formula

$$V=\frac{\mathrm{ADC}\times V_{\mathrm{ref}}}{4096},\;V_{\mathrm{ref}}=3.282V$$

These voltages are then translated into resistance values using a voltage divider model, where the thermistor is connected in series with a known 5.05 megaohm resistor. The resistance *R* is computed as

$$R=\left(\frac{2.99-V}{V}\right)\times 5.05\times 10^{6}$$

Only valid readings with 0 < *V* < 2.99 V are accepted for further processing. Once resistance values are obtained, they are normalized by their initial reference values *R_0_* (measured in the first frame). The temperature *T* in degrees Celsius is then computed using a piecewise logarithmic model based on the ratio *R/R_0_*. Finally, the temperature is clipped to the range [−5°C, 50°C] to eliminate extreme outliers caused by sensor drift or signal noise. The entire data acquisition and processing pipeline is designed to operate in real time, ensuring timely feedback for robotic control applications. The system also includes error handling for malformed frames and ensures graceful shutdown upon interruption.

### Control algorithm for perception-guided actuation (robotic platform)

The control system integrates real-time temperature sensing with robotic hand manipulation in a perception-guided feedback loop. Initially, the robotic hand grasps and lifts the object while temperature data are continuously monitored. If the sensed temperature subsequently reaches the lower bound of −5°C (representing a critical low-temperature event), the sensed temperature falls below the predefined threshold of −5°C during this process, the system initiates the sequence for placing down and releasing the object, otherwise, the grasp is maintained. The robotic hand is controlled using a position-current hybrid proportional–integral–derivative (PID) scheme with carefully tuned proportional (KP), integral (KI), and derivative (KD) gains to balance responsiveness and stability. Current limits are imposed to prevent motor overheating and excessive force. Joint positions are updated iteratively, considering joint stability over recent time steps to refine the grasp posture safely. Commands for the robotic arm’s movements are issued asynchronously to a separate control node responsible for arm positioning. This architecture enables seamless integration of sensory perception and actuation. The implementation is realized in Python, leveraging ROS middleware and ZeroMQ for inter-process communication, ensuring responsive and robust control.

## Supplementary Materials

### The PDF file includes:

Texts S1 to S5 Figs. S1 to S28 Table S1 Legends for movies S1 and S2
