# Supplementary material for: Bioinspired supramolecular thermistor for resolving subtle temperature variations
Source: Sci Adv. 2026 Oct 14;12(42):eaei0002. doi: 10.1126/sciadv.aei0002 (PMC13665936; doi:10.1126/sciadv.aei0002)
Supplement: Supplementary file 1 [file sciadv.aei0002_sm.pdf]

Science Advances
AAAS

Supplementary Materials for

Bioinspired supramolecular thermistor for resolving subtle temperature variations

Yangtao Yu *et al.*

Corresponding author: Yahui Li, yahui.li@ntu.edu.sg; Hesheng Wang, wanghesheng@sjtu.edu.cn; Zhuoqing Yang, yzhuoqing@sjtu.edu.cn; Yuanjin Zheng, yjzheng@ntu.edu.sg

Sci. Adv. **12**, eaei0002 (2026)
DOI: 10.1126/sciadv.aei0002

The PDF file includes:

- Texts S1 to S5
- Figs. S1 to S28
- Table S1
- Legends for movies S1 and S2

Other Supplementary Material for this manuscript includes the following:

- Movies S1 and S2

Supplementary Text

Text S1. Biological thermal transduction through temperature-gated ion channels

Thermal perception in biological systems begins at the sensory interface, where specialized thermoreceptors convert temperature changes into electrical signals. Biological thermal sensing is achieved through temperature-gated ion channels, whose molecular conformational changes regulate ion permeability in response to thermal stimuli. This molecular gating process provides the fundamental mechanism by which temperature information is encoded into electrical activity. Temperature-dependent ion channels are embedded in the membranes of peripheral sensory neurons. Upon cooling, thermal energy modifies the conformational equilibrium of these membrane proteins, increasing the probability that the ion-conducting pore adopts an open state. The resulting influx of cations, particularly Na^+ and Ca^{2+}, depolarizes the membrane and generates a receptor potential. When this depolarization exceeds the excitation threshold, voltage-gated sodium channels are subsequently activated, initiating action potentials that propagate along sensory axons toward the central nervous system for thermal perception.

Inspired by this general biological principle rather than any specific receptor subtype, our material system emulates the concept of molecularly gated thermal transduction. Instead of protein conformational changes regulating membrane ion channels, temperature induces dynamic reconfiguration of a supramolecular hydrogen-bond network, which continuously modulates coupled ionic and electronic transport within the composite. Although the underlying materials and transport mechanisms differ fundamentally from biological ion channels, both systems share the common design principle that temperature-dependent molecular structural evolution governs charge transport and ultimately determines the electrical output.

Text S2. Analysis of thermally activated ionic and electronic transport

The temperature-dependent electrical response of the supramolecular thermistor originates from the cooperative contribution of thermally activated electronic transport in NiO and hydrated ionic transport within the CMC-based hydrogen-bond network. In both cases, the temperature dependence of resistance can be approximately described by the Arrhenius relationship:

$$R(T) = R_0 \exp\left(\frac{E_a}{k_B T}\right)$$

Where $R(T)$ is the resistance at temperature T, R_0 is the pre-exponential factor corresponding to the high-temperature limiting resistance, E_a is the apparent activation energy, and k_B is the Boltzmann constant.

For the electronic component, NiO behaves as a typical p-type semiconductor, where charge transport is dominated by hole conduction associated with the Ni^{2+}/Ni^{3+} redox equilibrium and intrinsic defects such as nickel or oxygen vacancies. As temperature increases, thermal excitation promotes holes generation and transport, leading to enhanced electronic conductivity and a corresponding decrease in resistance. Meanwhile, ionic conduction primarily originates from hydrated ions within the CMC matrix, including Na^+ and H_3O^+ species. Their migration is also thermally activated and therefore follows Arrhenius-type behavior. Increasing temperature enhances ionic mobility by providing sufficient thermal energy to overcome migration barriers, resulting in reduced ionic resistance.

Unlike conventional ionic conductors, however, ion migration in the present supramolecular system is strongly coupled with the dynamic evolution of the hydrogen-bond network. Thermal activation promotes segmental motion of CMC chains and continuous reorganization of hydrogen bonds, which dynamically regulates ion-conductive pathways. Hydrated ions migrate through transient hydrogen-bond-assisted hopping between electronegative functional groups such as hydroxyl and carboxylate moieties. Consequently, the apparent activation energy obtained from Arrhenius analysis reflects not only ion migration but also the concurrent restructuring of the supramolecular network. Therefore, the overall thermoresistive response of the composite cannot be attributed solely to either electronic or ionic transport. Instead, it arises from the cooperative evolution of thermally activated hole conduction in NiO together with hydrogen-bond-regulated ionic transport within the hydrated CMC network.

Text S3. Crystallinity analysis of Ni-based compounds at different temperatures

The XRD patterns at all measured temperatures exhibit prominent diffraction peaks at 2θ = 37.32°, 43.36°, 63.00°, 75.56°, and 79.57°, which correspond to the (111), (200), (220), (311), and (222) planes of NiO (fig. S3), consistent with the standard JCPDS No. 04-002-5335. At lower temperatures (-30 °C and -45 °C), additional diffraction peaks appear, attributed to $Ni(OH)_2 \cdot 0.75H_2O$ (JCPDS No. 00-038-0715), $Ni(OH)_2 \cdot 2H_2O$ (JCPDS No. 00-022-0444), and NiOOH (JCPDS No. 00-006-0075), indicating temperature-dependent phase transitions.

To evaluate the overall crystallinity of the composite, the following equation was employed:

$$X = \frac{A_{crystalline}}{A_{crystalline} + A_{amorphous}} \times 100\%$$

The calculated crystallinity of the composite at 15 °C, 0 °C, -15 °C, -30 °C, and -45 °C were 95.95%, 95.63%, 94.71%, 94.00%, and 92.47%, respectively. These results reveal a clear trend of decreasing crystallinity with the temperature decreases.

Text S4. Analysis of thermal hysteresis

To quantify the thermal hysteresis behavior of the sensor, we analyzed the normalized resistance values (R/R_0) during both the heating and cooling cycles at the same temperature points. The hysteresis ratio is calculated using the formula:

$$Thermal\ hysteresis = \frac{R_{heating} - R_{cooling}}{R_{heating}}$$

Then, we counted the deviation values corresponding to all temperature points.

Text S5. Rationale for the tropospheric temperature setting

The programmed cooling rate is consistent with typical atmospheric lapse conditions during balloon ascent. In the troposphere, temperature generally decreases at an average lapse rate of approximately 6-6.5 °C km^{-1}. A temperature drops from 20 °C to -60 °C corresponds to an altitude change of roughly 12 km, which is comparable to the typical height of the tropopause. Assuming a representative meteorological balloon ascent velocity of 4-6 m s^{-1}, reaching this altitude requires approximately 30-45 min, yielding an effective cooling rate on the order of ~2 °C min^{-1}. Therefore, the programmed temperature trajectory provides a reasonable laboratory approximation of the thermal conditions experienced during balloon ascent through the troposphere.

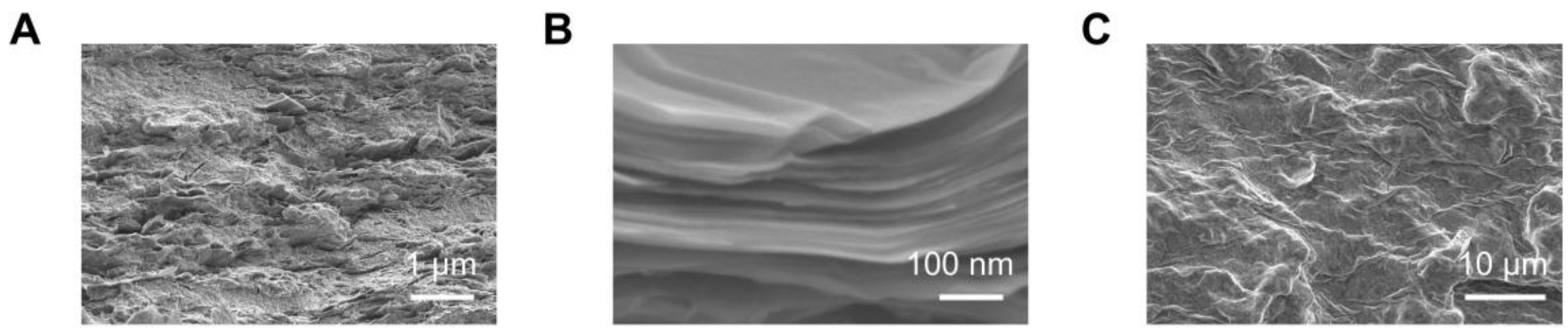

Fig. S1. SEM images of the synthesized composites. (**A**) Low-magnification image of the CMC/MXene/NiO composite showing packed, irregular surface morphology with limited interfacial coherence. (**B**) High-magnification image of the CMC/MXene/NiO composite, revealing partially aligned layered structures. (**C**) Supramolecularly integrated PU/CMC/MXene/NiO composite, exhibiting densely entangled fibrous architecture.

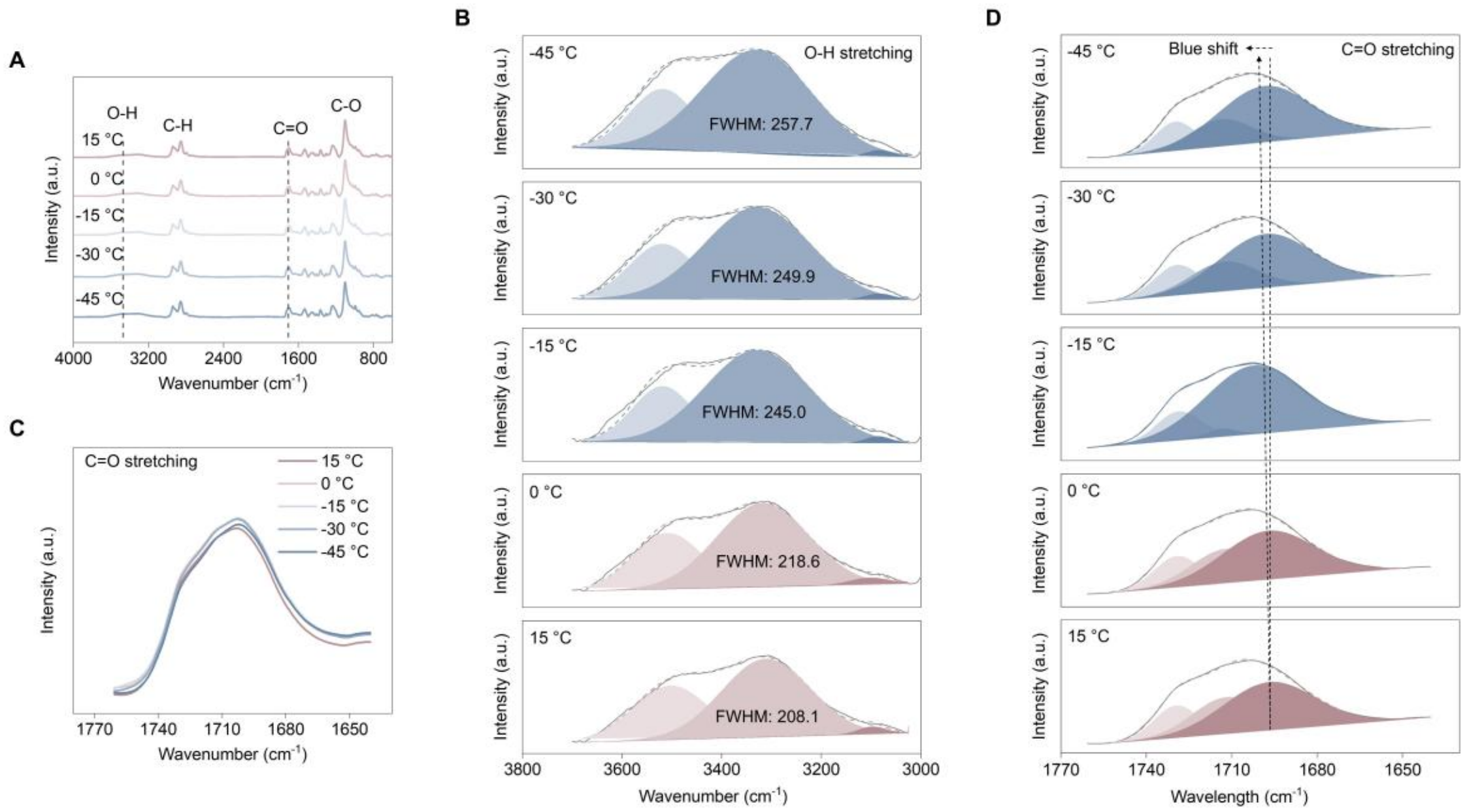

Fig. S2. In-situ FTIR analysis of hydrogen-bond-related vibrational modes. (**A**) Temperature-dependent FTIR spectra. (**B**) Evolution of the hydrogen-bonded O-H component with increasing FWHM at lower temperatures. (**C**) Temperature-dependent evolution of the C=O stretching band. (**D**) Deconvolution of the C=O stretching band, showing a temperature-dependent blue shift upon cooling.

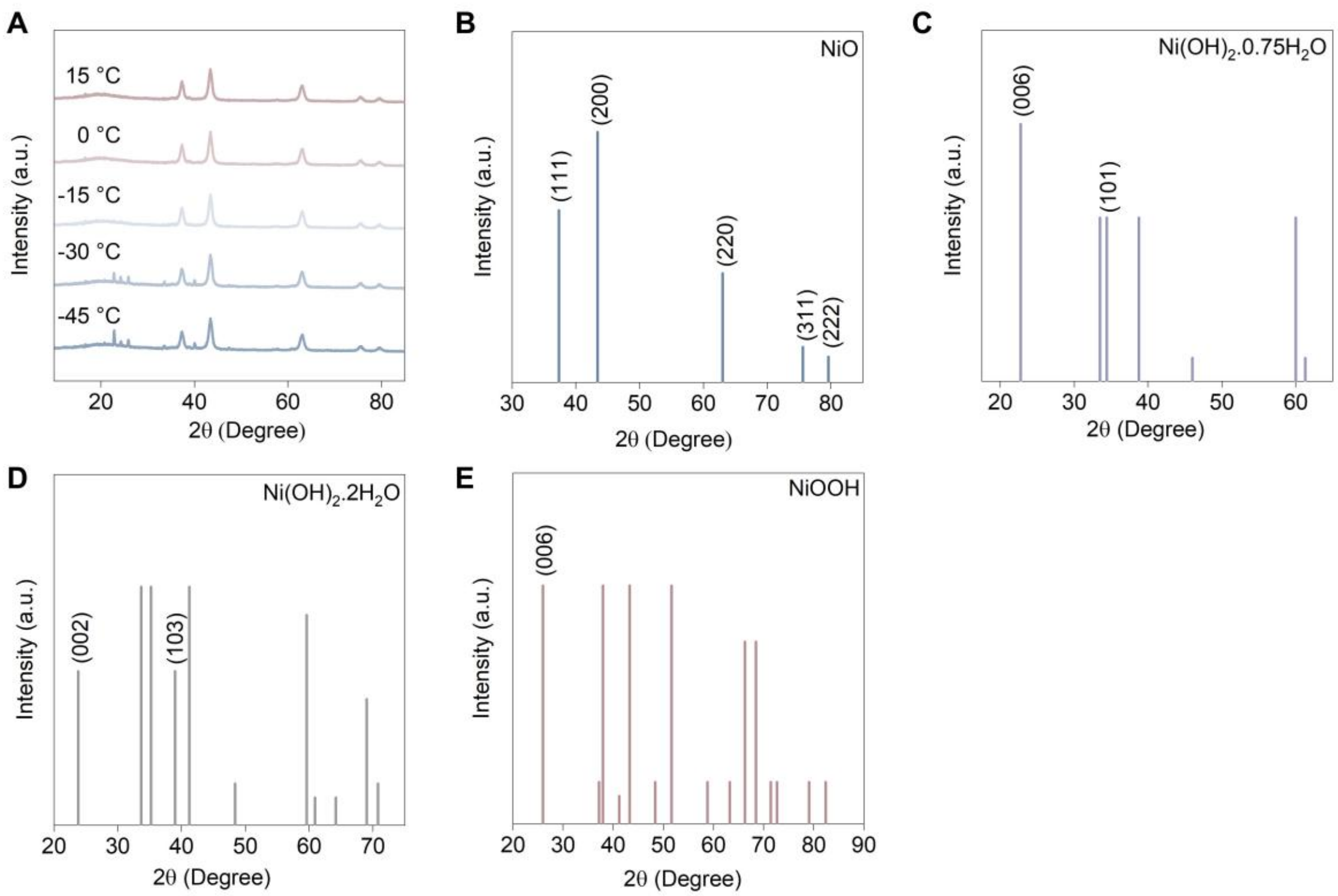

Fig. S3. Temperature-dependent XRD spectra and reference phase identification. (**A**) XRD spectra of the PU/CMC/MXene/NiO composite recorded from 15 °C to -45 °C. (**B-E**) Reference diffraction patterns of nickel-containing phases: (**B**) NiO, (**C**) $Ni(OH)_2 \cdot 0.75H_2O$, (**D**) $Ni(OH)_2 \cdot 2H_2O$, and (**E**) NiOOH.

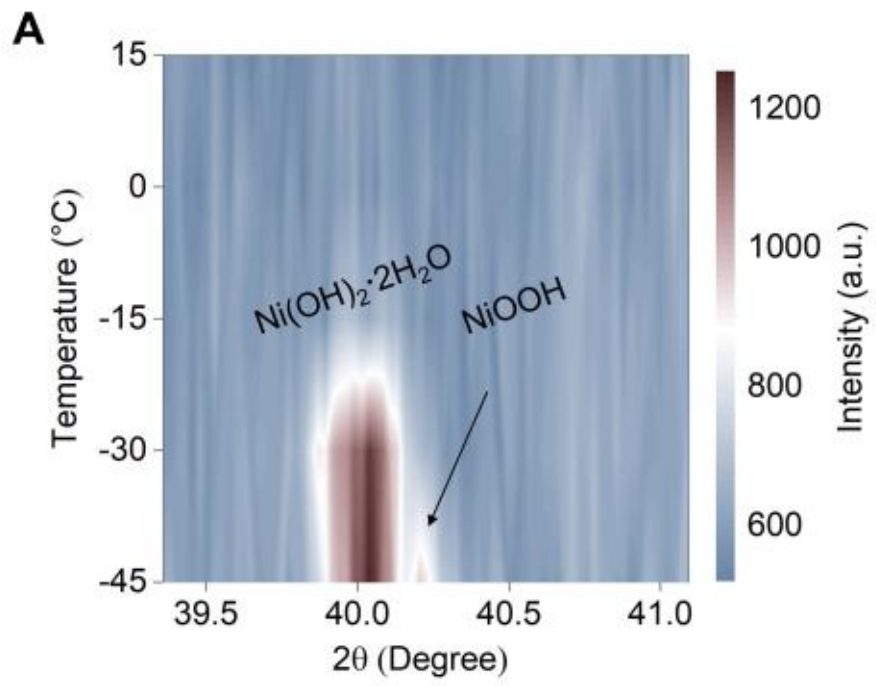

Fig. S4. In-situ XRD mapping of NiO phase transformation and hydration state transitions. (**A**) Diffraction mapping at 2θ ≈ 40°, highlighting the emergence and transition between $Ni(OH)_2 \cdot 2H_2O$ and NiOOH phases with decreasing temperature. (**B**) Diffraction mapping at 2θ ≈ 33.6°, corresponding to the progressive formation of $Ni(OH)_2$ as temperature drops below -15 °C.

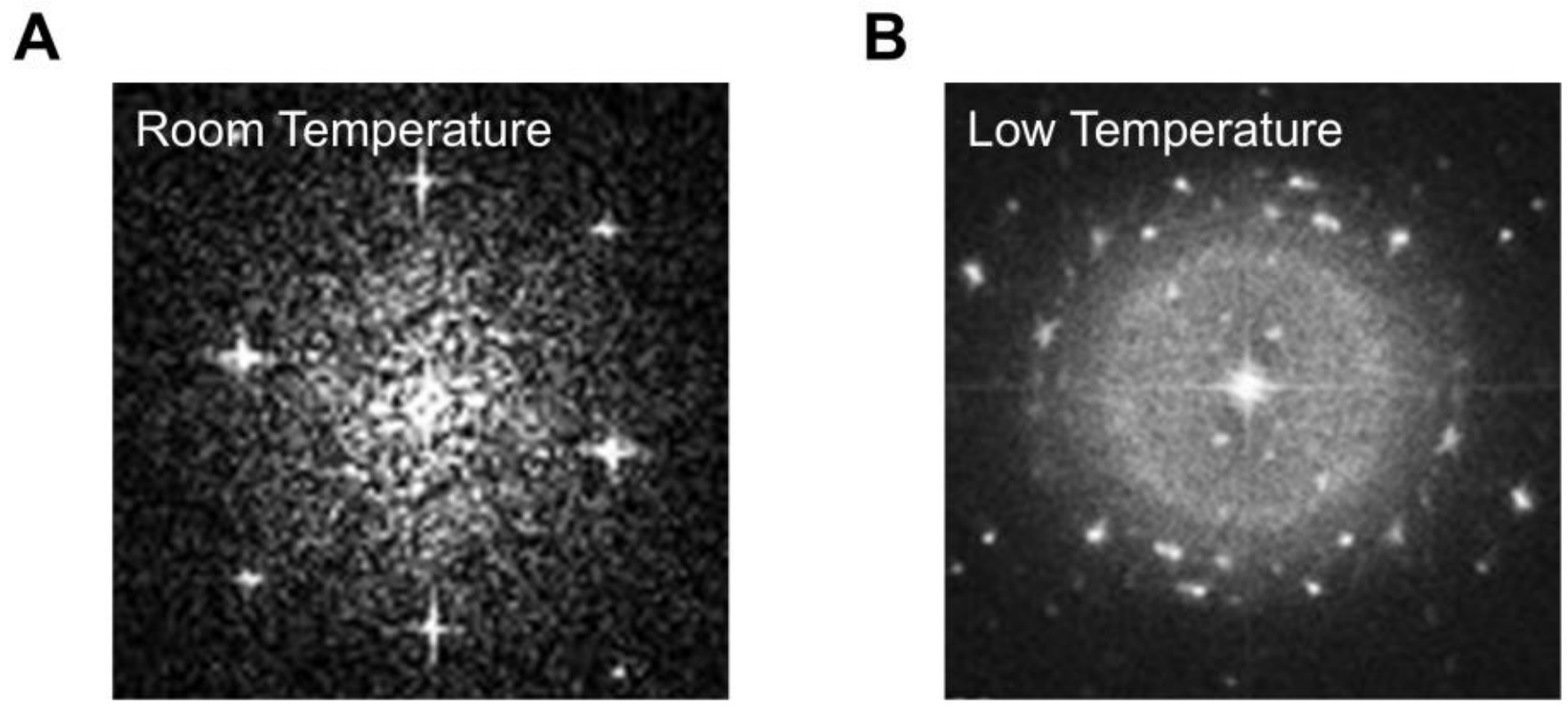

Fig. S5. Selected-area electron diffraction (SAED) patterns of NiO-based phases at different temperatures. (**A**) SAED pattern at room temperature, showing distinct and sharp diffraction spots corresponding to well-crystallized NiO domains. (**B**) SAED pattern at low temperature, exhibiting broadened and diffuse diffraction rings, indicating partial phase transformation and the formation of hydrated or hydroxylated nickel species such as $Ni(OH)_2 \cdot 2H_2O$ and NiOOH.

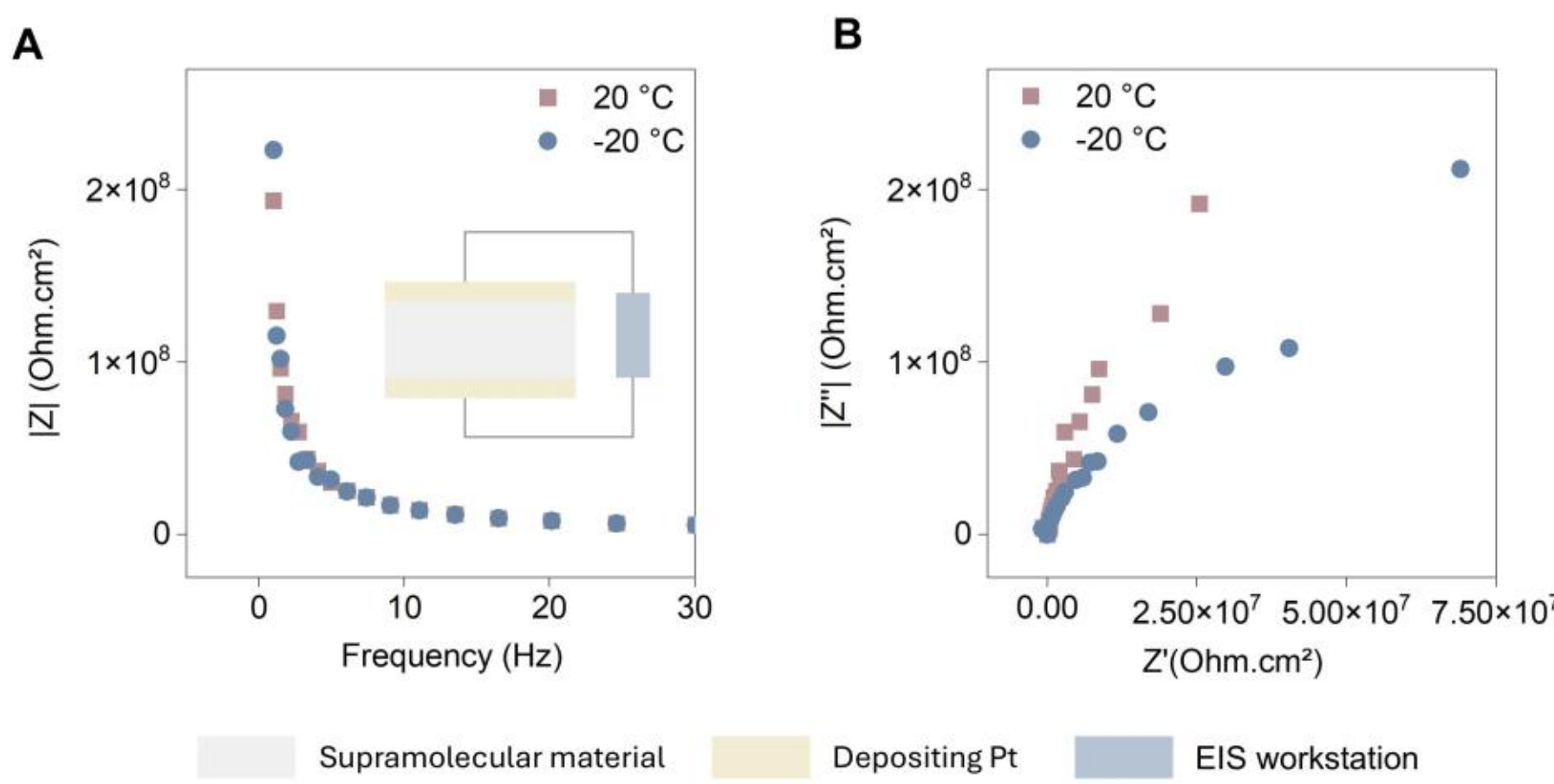

Fig. S6. Temperature-dependent EIS characterization of charge transport in the supramolecular thermistor. (**A**) Frequency-dependent impedance spectra at 20 °C and -20 °C with the EIS measurement configuration shown in the inset. (**B**) Corresponding Nyquist plots, indicating increased transport resistance at lower temperatures.

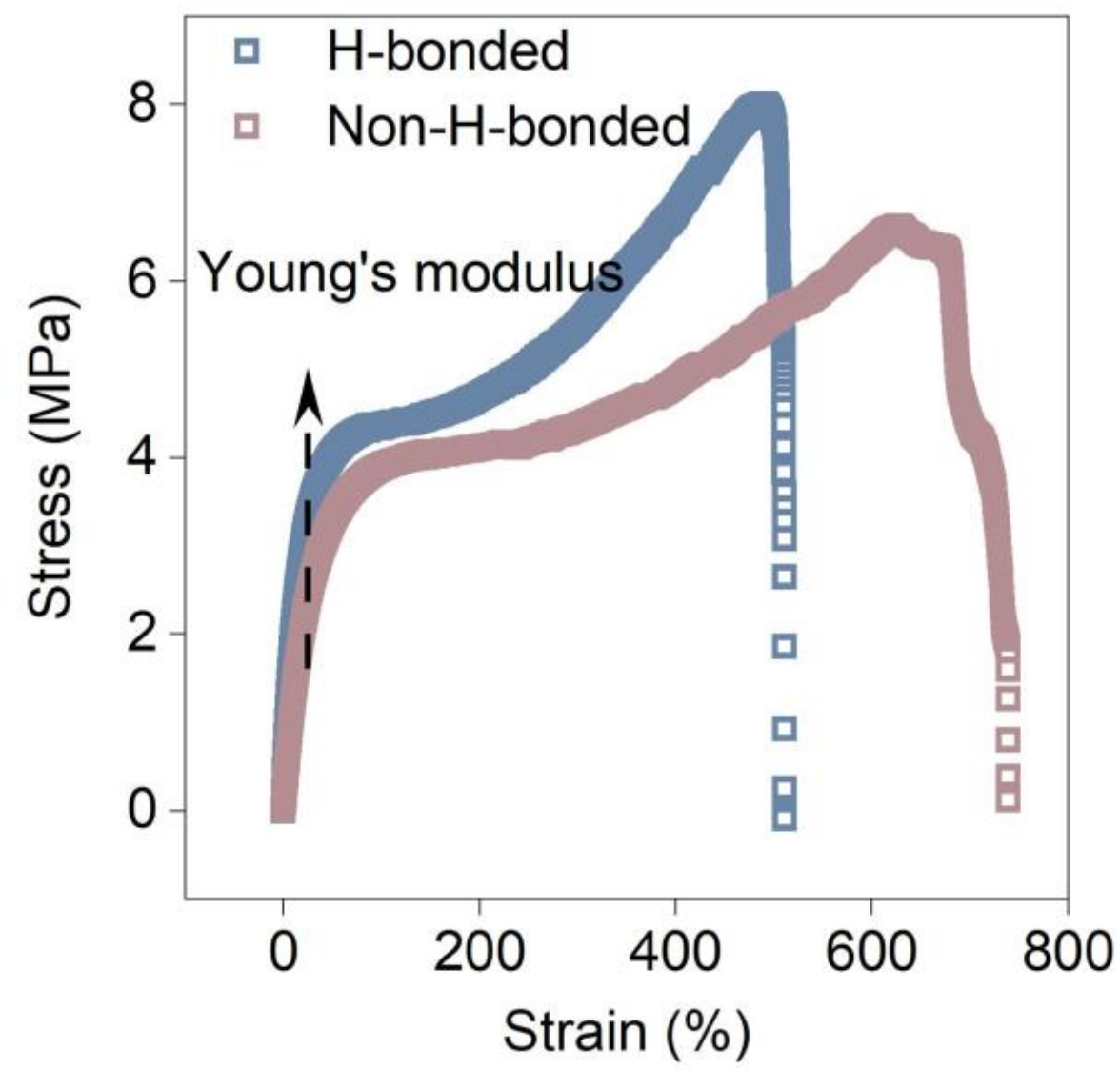

Fig. S7. Comparison of tensile properties between H-bonded and non-H-bonded composites. Stress-strain curves of H-bonded composite and non-H-bonded control sample showing differences in Young's modulus, tensile strength, and stretchability.

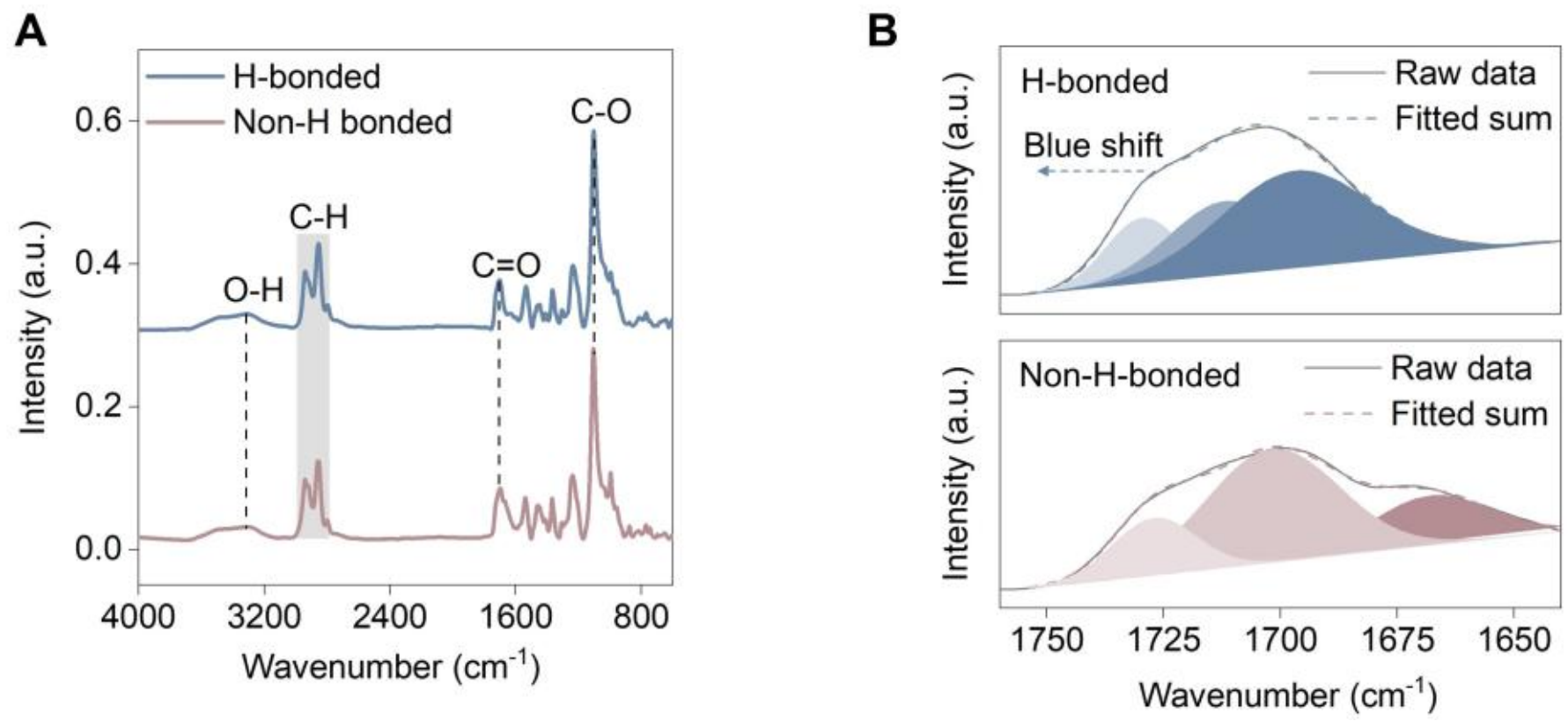

Fig. S8. FTIR comparison between hydrogen-bonded and non-hydrogen-bonded composites. (**A**) Characteristic FTIR spectra of hydrogen-bonded and non-hydrogen-bonded composites. (**B**) Deconvoluted C=O stretching bands and blue shift in the hydrogen-bonded composite.

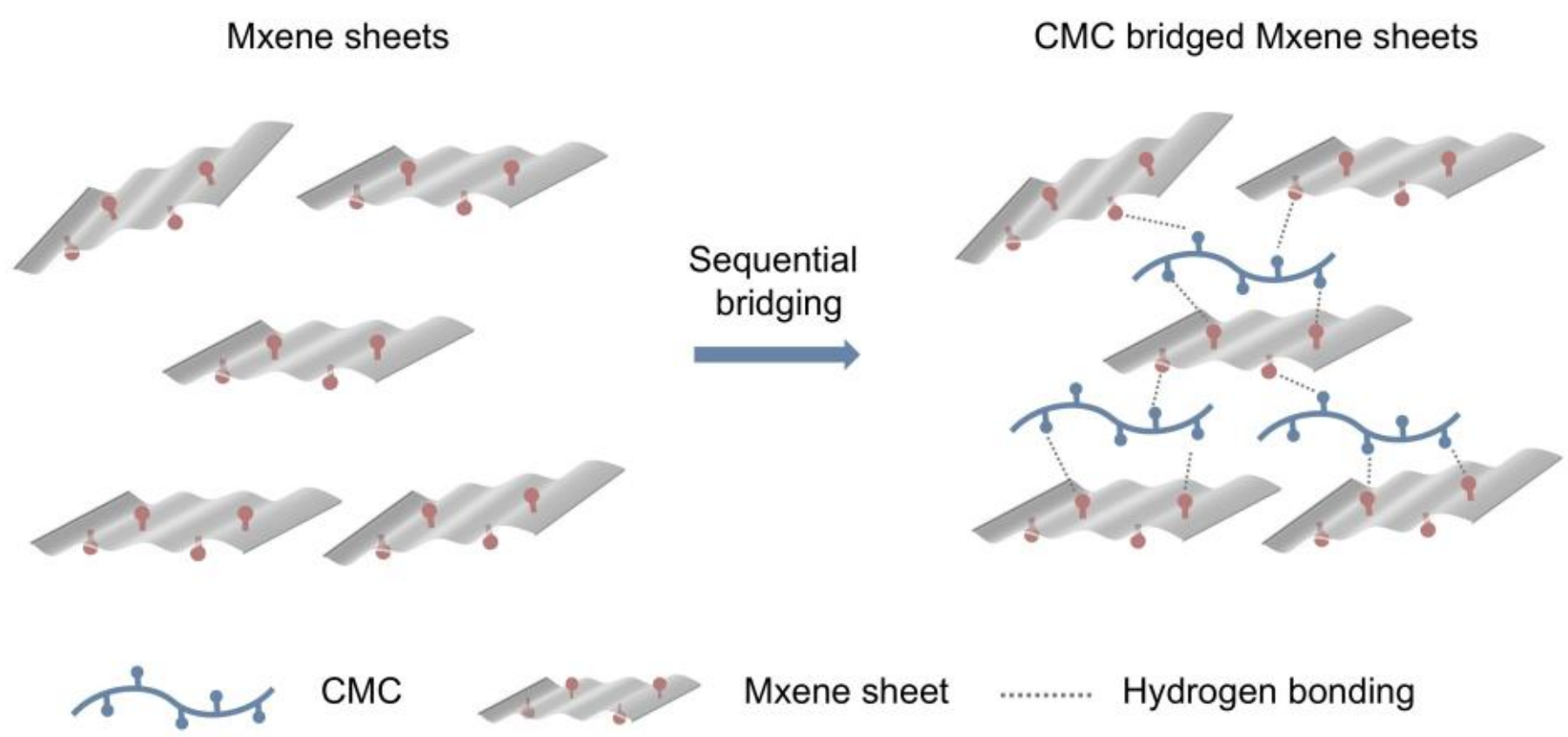

Fig. S9. Structural model of the MXene sheets bridged by CMC chains, indicating the formation of ordered and layered structure with hydrogen bonding.

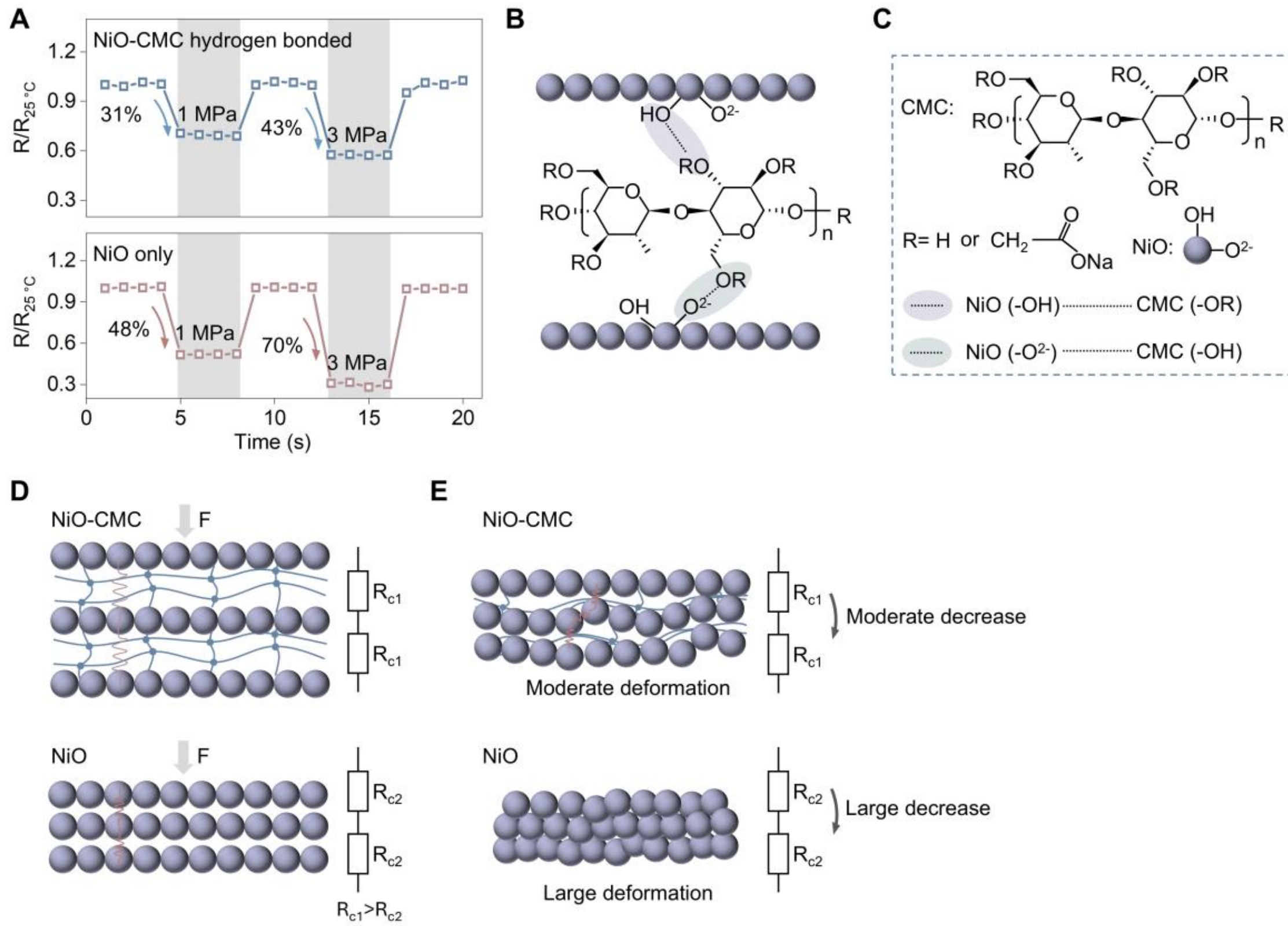

Fig. S10. Pressure-resistance behavior and hydrogen bonding in PU/CMC/NiO composite. (**A**) Resistance response of NiO-CMC composite and pure NiO under applied pressures (1 MPa and 3 MPa). (**B** and **C**) Schematic illustration of hydrogen bonding interactions between carboxymethyl cellulose (CMC) and NiO surface groups. (**D** and **E**) Structural models showing deformation and resistance variation under pressure.

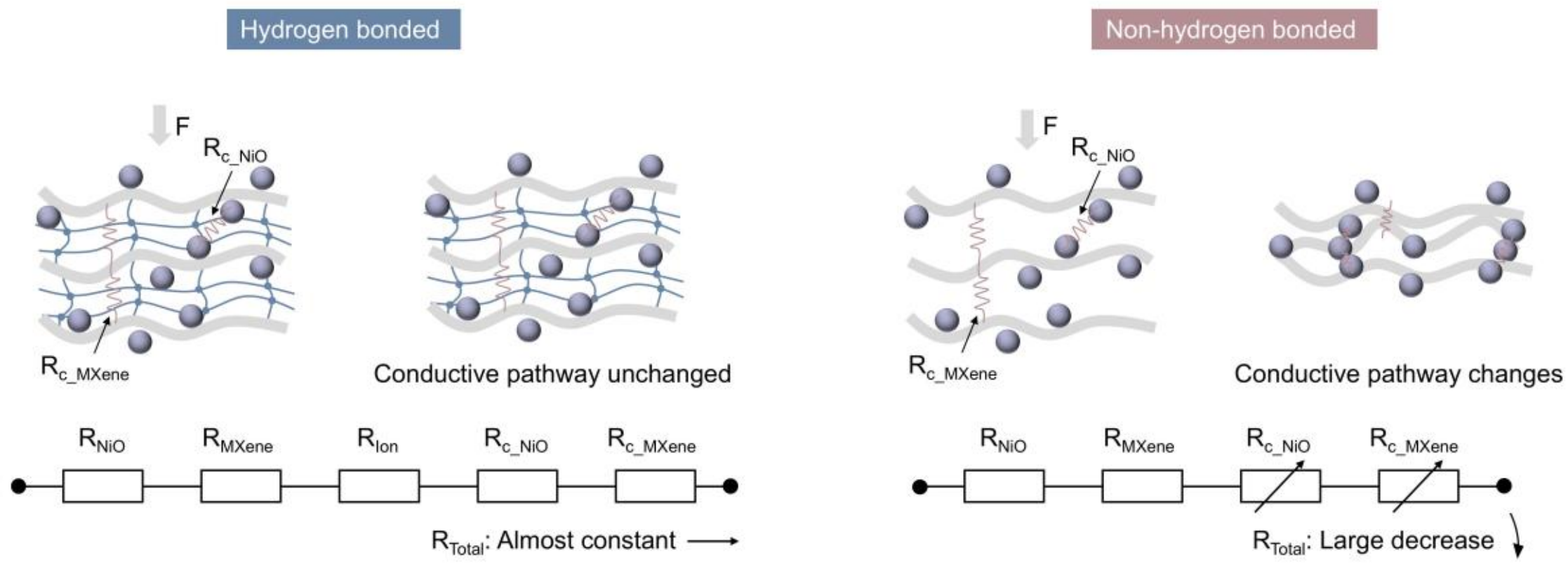

Fig. S11. Schematic illustration of hydrogen-bond-mediated mechanical stabilization of conductive pathways. Left: Hydrogen-bonded network with preserved conductive pathways and nearly constant total resistance under compression. **Right:** Non-hydrogen-bonded network with disrupted conductive pathways and reduced total resistance under compression.

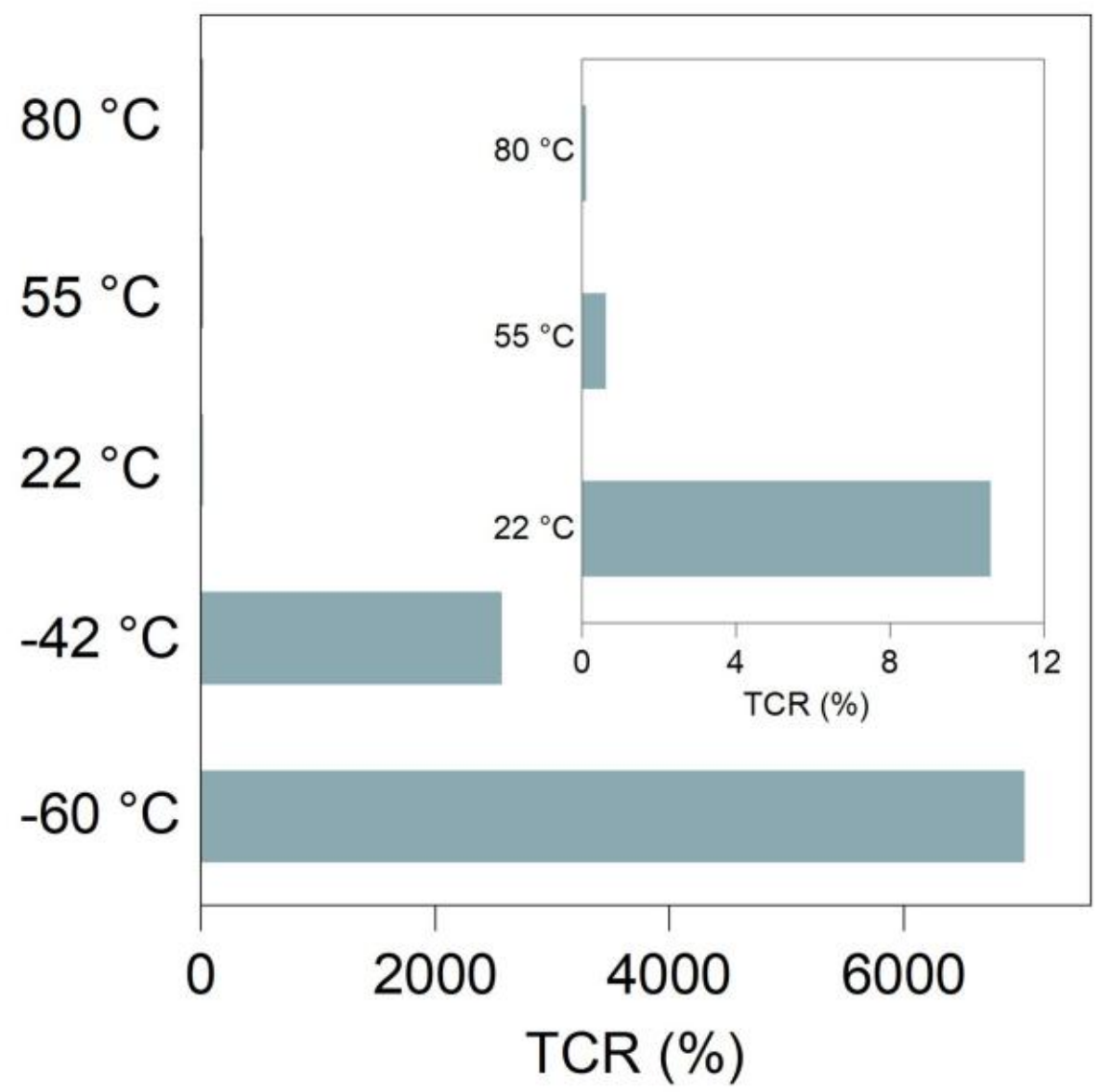

Fig. S12. Temperature coefficient of resistance (TCR) of the supramolecular thermal sensor measured from -60 °C to 80 °C. TCR values extracted across the full temperature range. The inset highlights the comparison in the higher-temperature regime (22 °C to 80 °C).

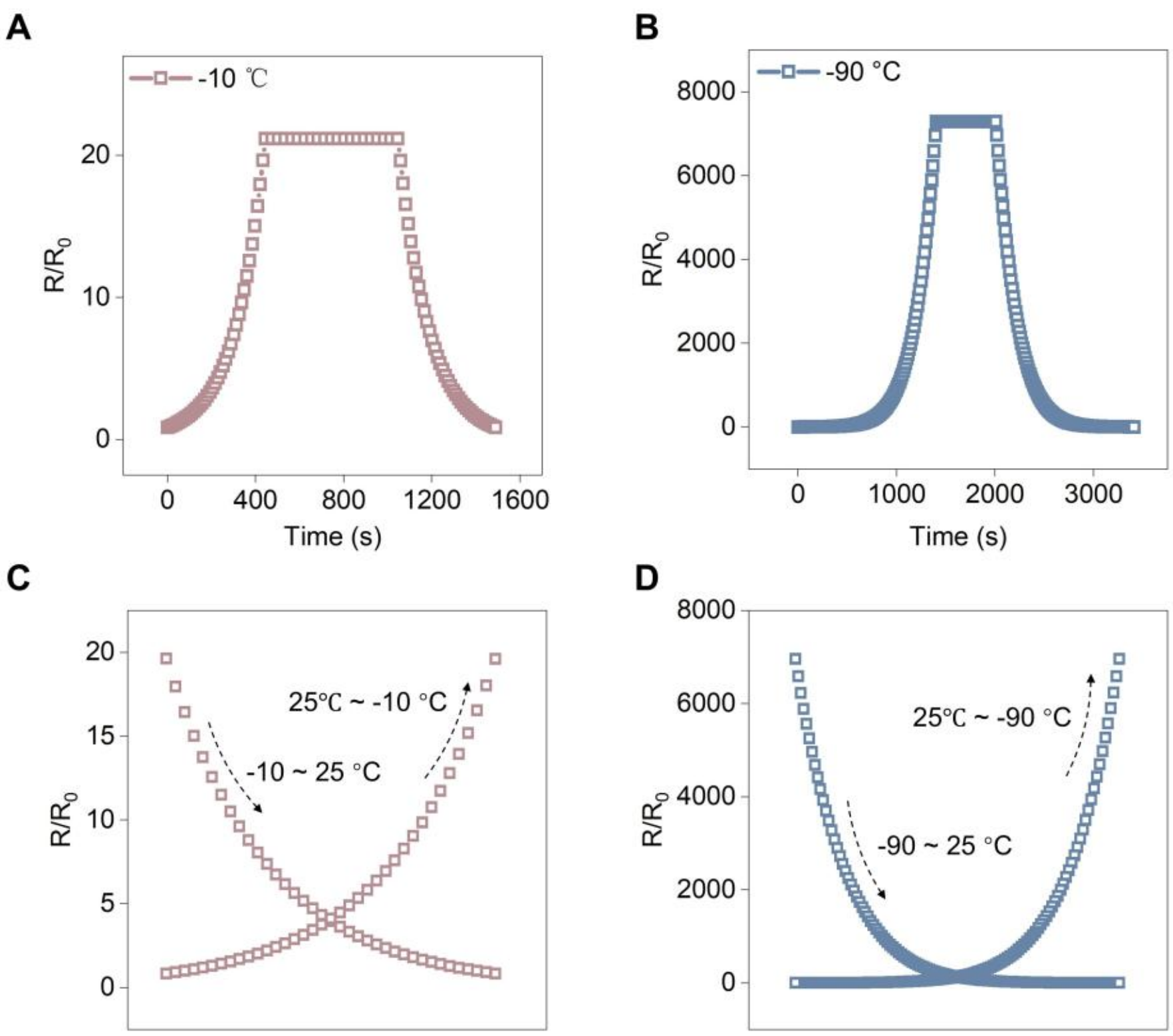

Fig. S13. Low-temperature stability and thermal cycling performance of the supramolecular thermistor. (**A**, **B**) Resistance response of the sensor during prolonged operation at -10 °C and -90 °C. (**C**) Thermal cycling measurement between 25 °C and -10 °C. (**D**) Thermal cycling measurement between 25 °C and -90 °C.

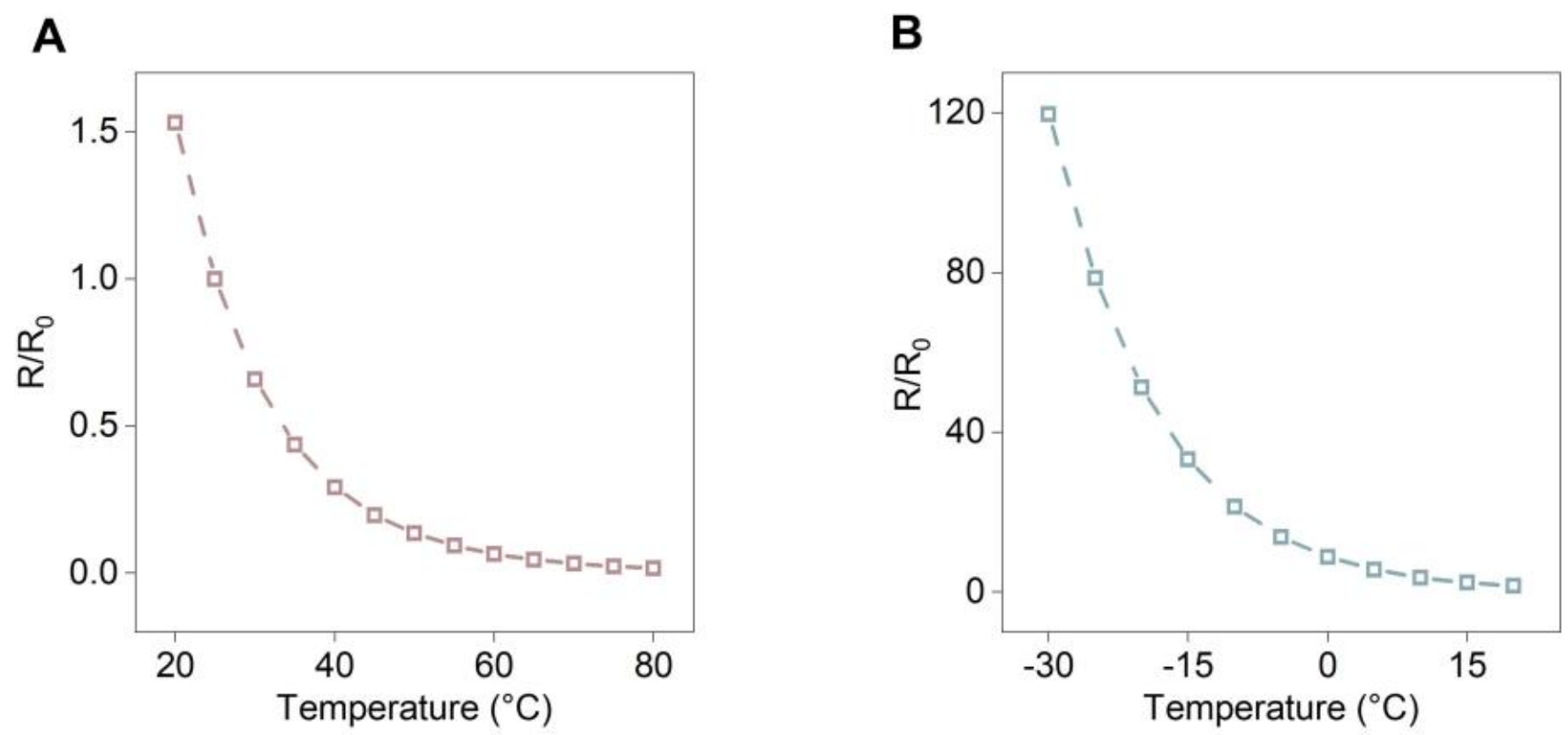

Fig. S14. Fitting curves of normalized resistance (*R/R_0*) over different temperature ranges. (**A**) Normalized resistance variation in the high-temperature range (20 °C to 80 °C). (**B**) Normalized resistance variation as a function of temperature in the low-temperature range (-30 °C to 20 °C).

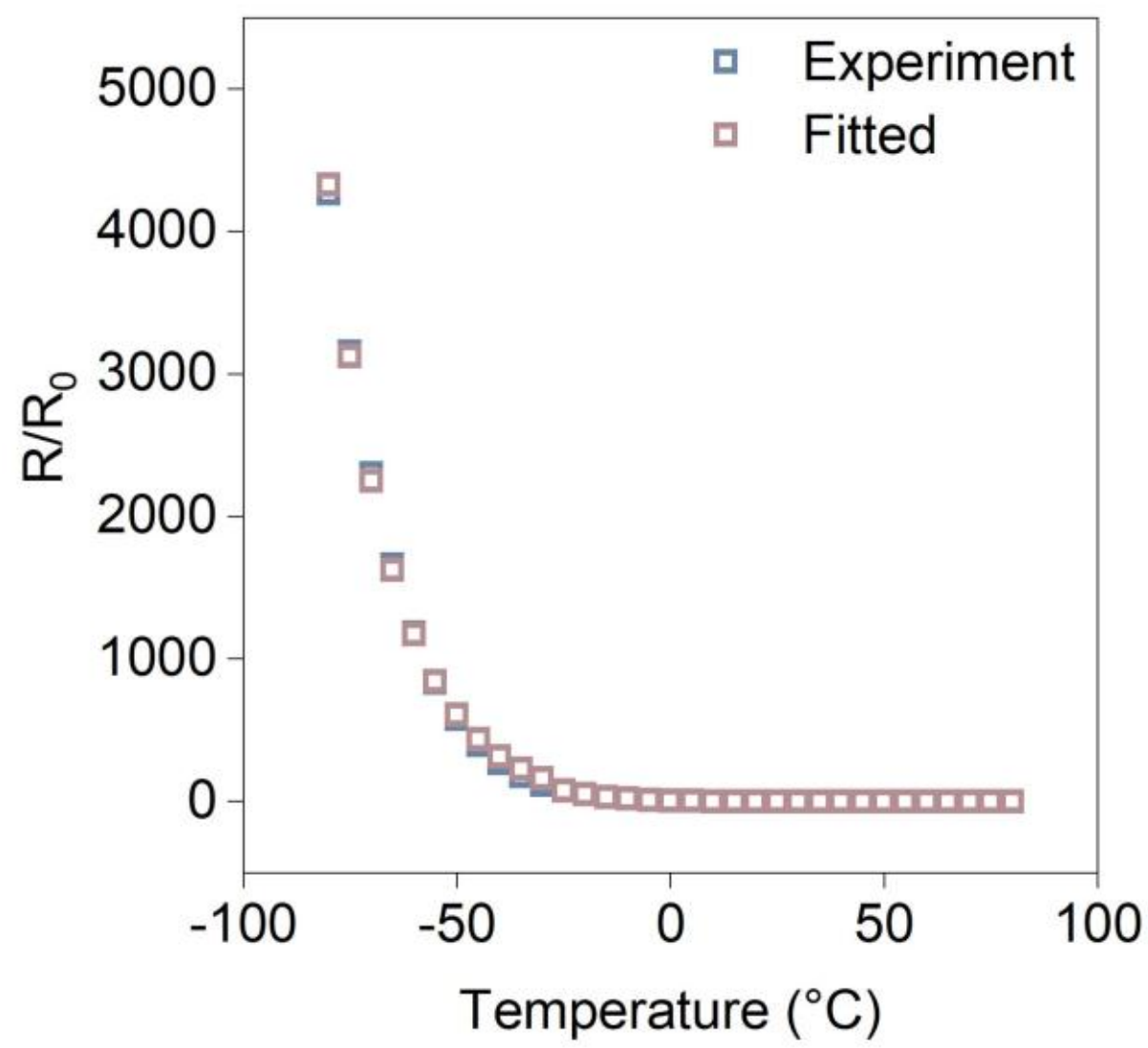

Fig. S15. Fitting curve validation against experimental data across a broad temperature range.

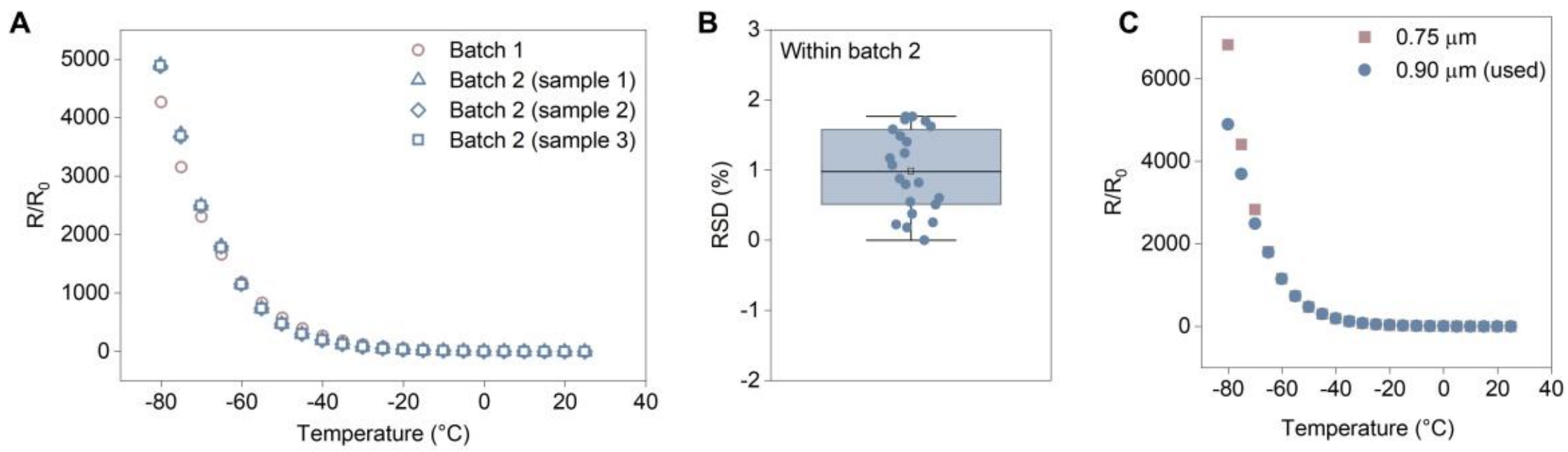

Fig. S16. Reproducibility of independently fabricated supramolecular thermistors. (**A**) Comparison of the normalized temperature-dependent resistance (R/R_0) measured from sensors fabricated in two independent batches. (**B**) Statistical analysis of the relative standard deviation (RSD) of multiple independently fabricated sensors within Batch 2. (**C**) Thickness-dependent thermoresistive responses (0.75 and 0.90 μm).

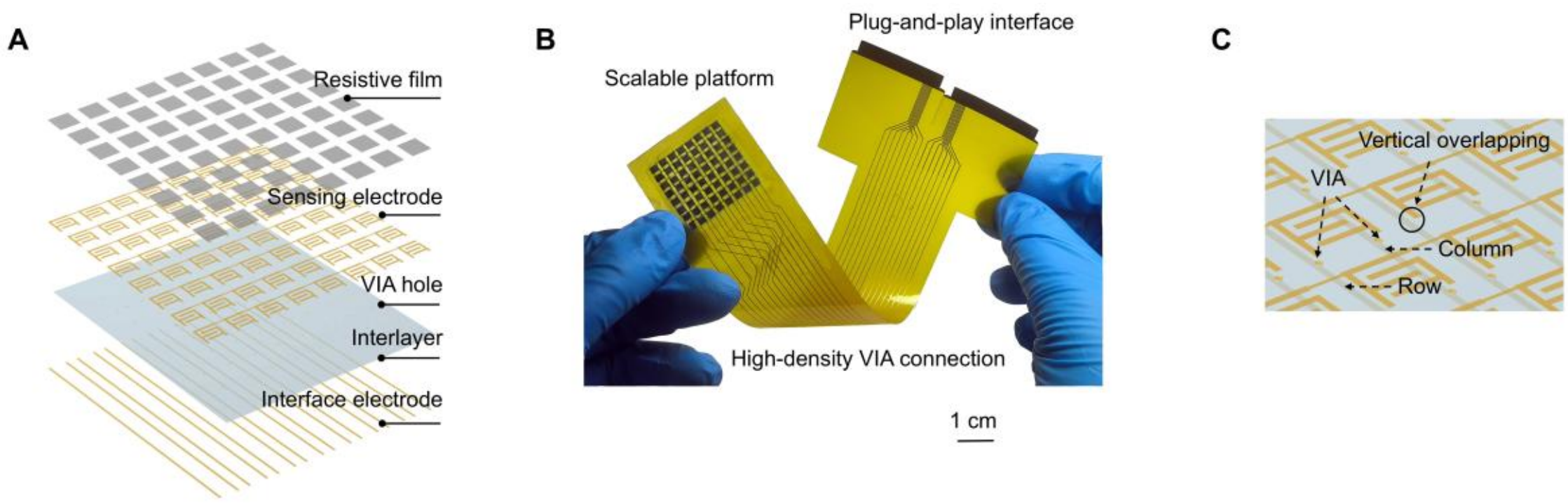

Fig. S17. Multilayer structural design and physical demonstration of the flexible sensing array. (**A**) Schematic illustration of the multilayer configuration of the sensor array, consisting of a resistive film, sensing electrode layer, interlayer dielectric, and interface electrode layer. The structure integrates vertical interconnect access (VIA) holes, enabling high-density electrical connections between stacked layers and ensuring compact, high-resolution sensing nodes. (**B**) Optical photographs of the fabricated flexible sensor array. The device exhibits excellent mechanical flexibility and structural extensibility, highlighting its potential for conformal integration and scalable sensing coverage in wearable or adaptive electronic systems. (**C**) Detailed electrode wring method with VIA structure.

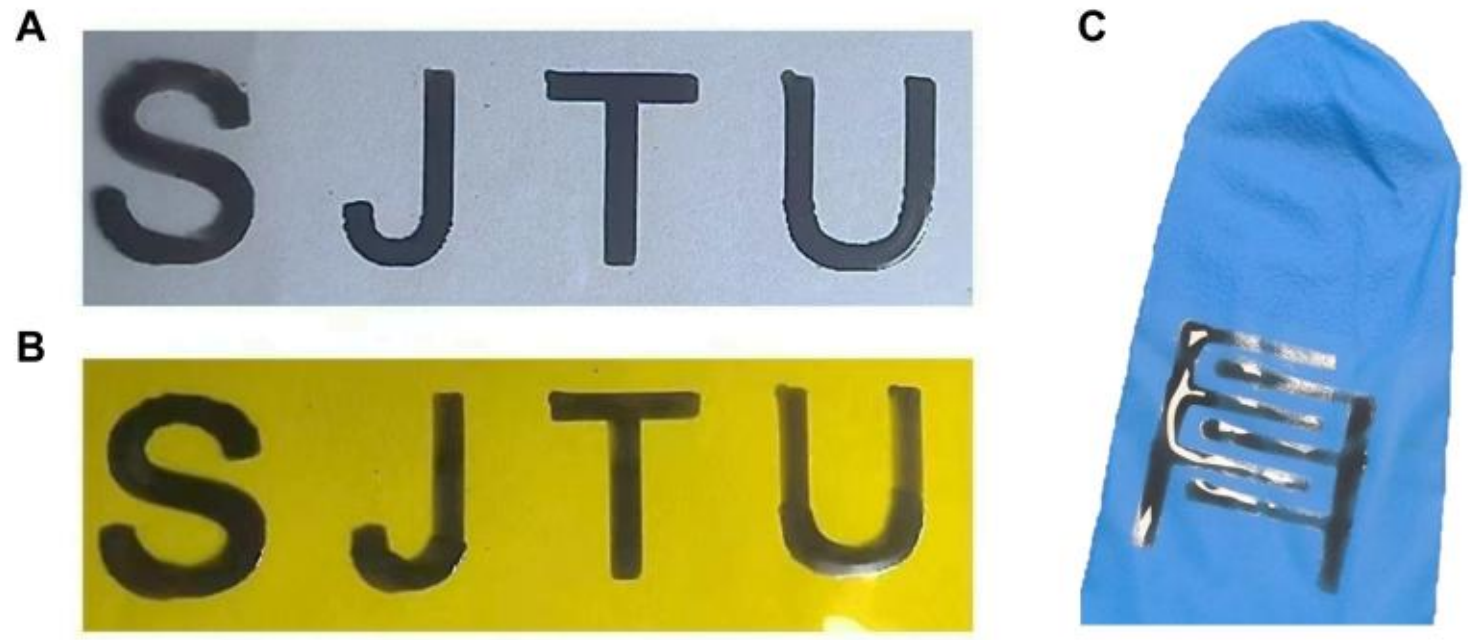

Fig. S18. Pattern fidelity and substrate compatibility of the printed supramolecular sensing film. Inkjet-printed “SJTU” patterns formed on different substrates: (**A**) polyethylene terephthalate (PET), (**B**) polyimide (PI), and (**C**) nitrile glove.

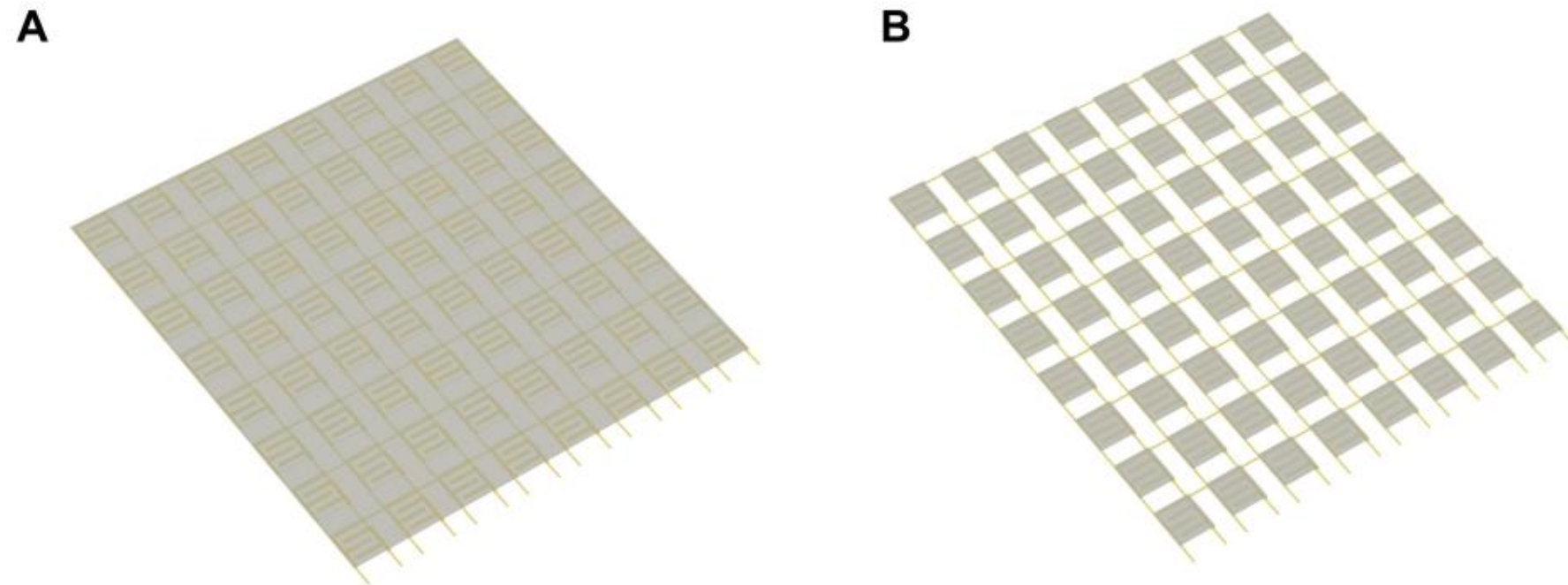

Fig. S19. Structural designs for different sensing configurations. (**A**) Uniform sensing layout where the sensing material forms a continuous, uniformly distributed film. (**B**) Discrete sensing layout, featuring an island-bridge configuration where sensing regions are spatially separated, suitable for localized detection.

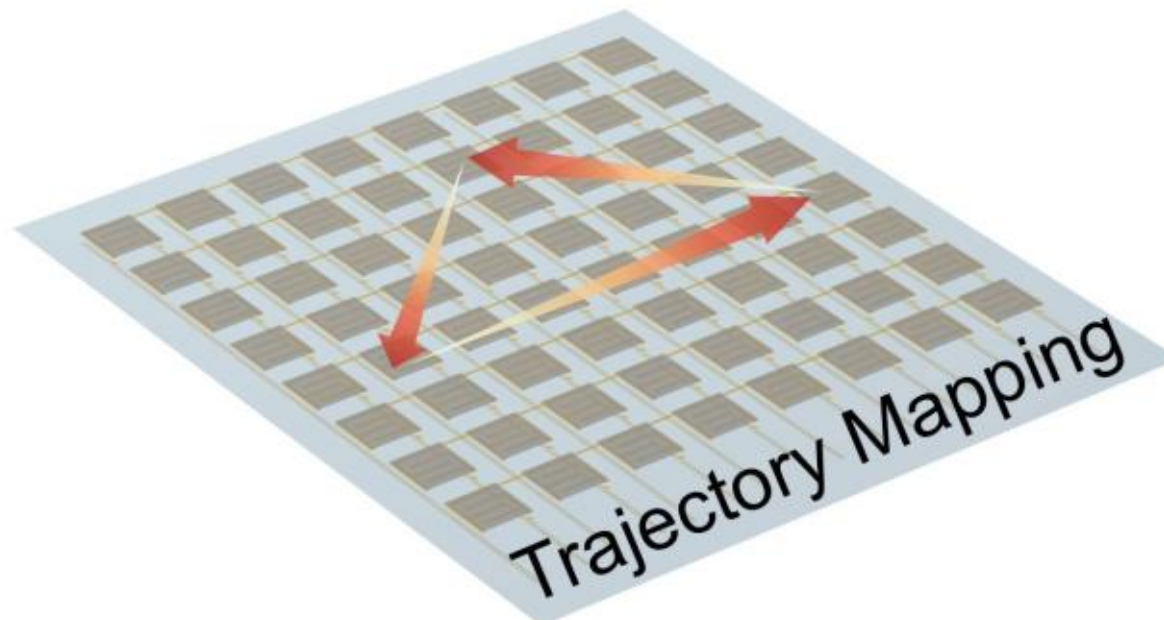

Fig. S20. Functional diagram showing trajectory mapping and object tracking enabled by the large-area sensor system.

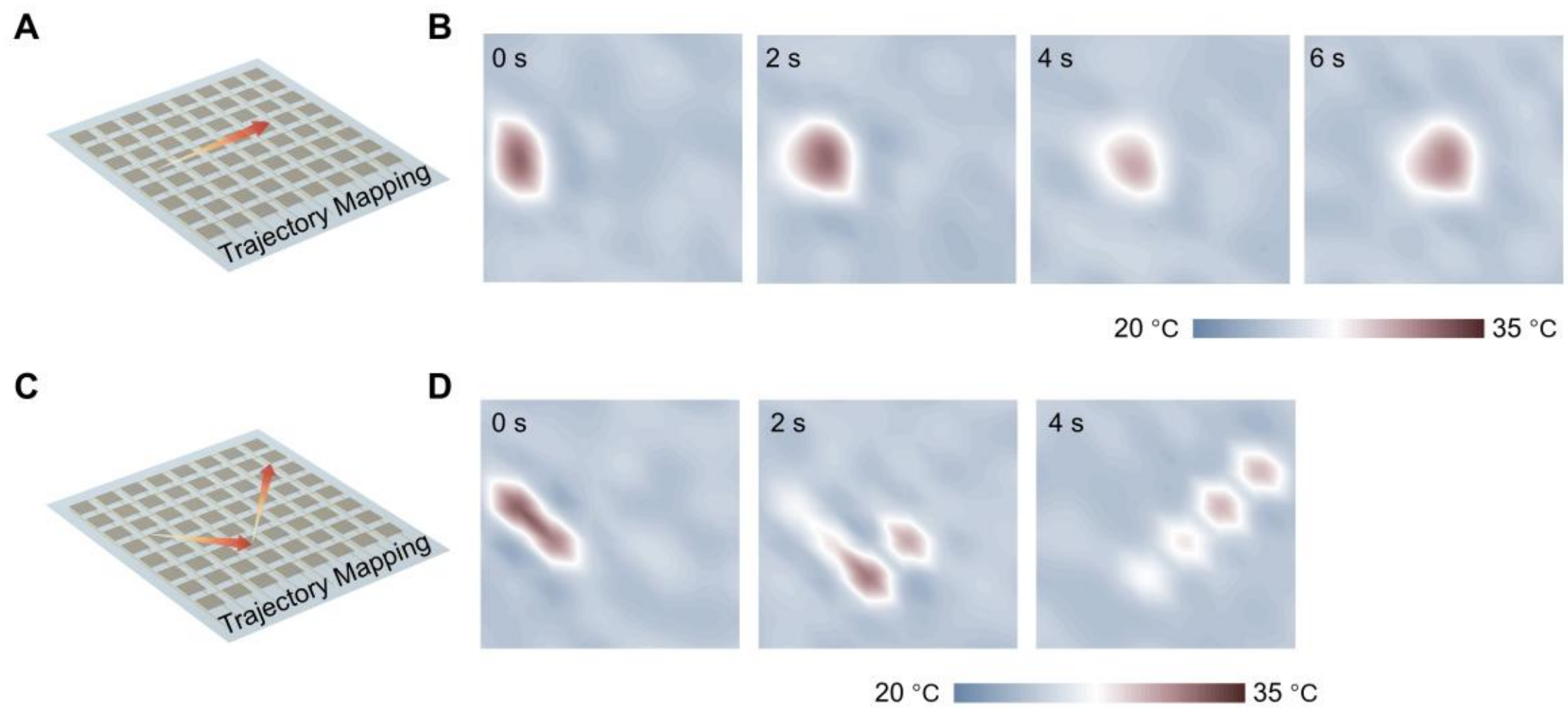

Fig. S21. Trajectory mapping of dynamic temperature stimuli on the sensing array. (**A** and **C**) Schematic illustrations showing directional movement paths of finger across the temperature sensor array. (**B** and **D**) Corresponding thermal mapping recorded at sequential time points, demonstrating the spatiotemporal temperature distribution.

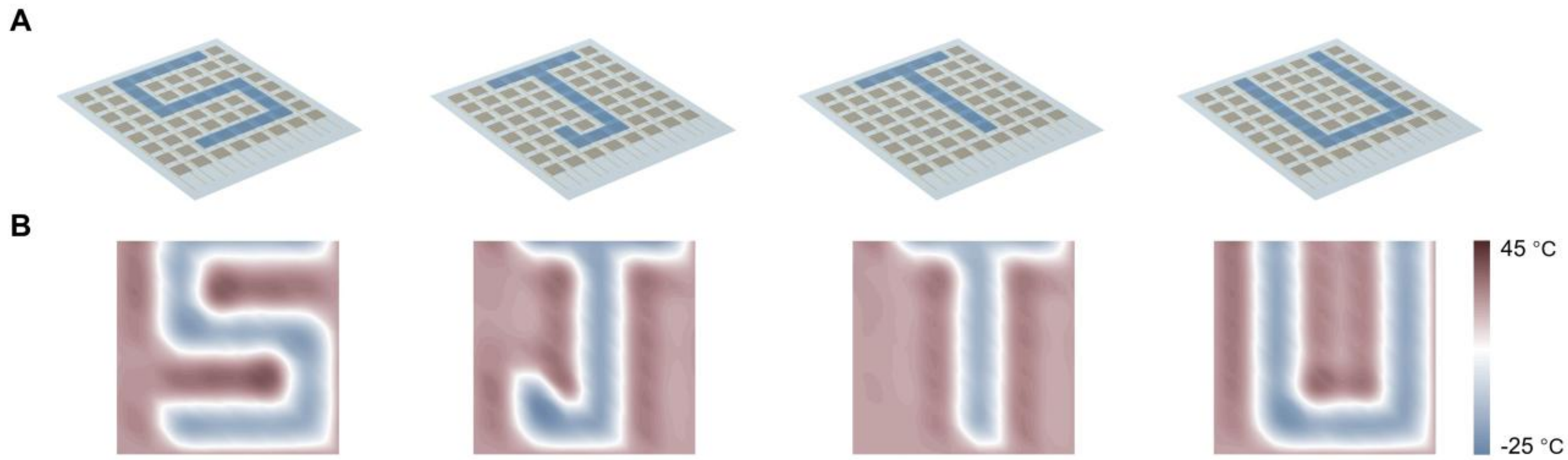

C

24.3	24.5	-12.4	-12.3	-12.0	-12.4	-12.1	25.0
24.6	24.4	-12.8	24.1	25.1	25.1	25.1	24.9
24.7	25.0	-11.9	25.1	24.3	25.1	24.2	25.1
25.0	24.2	-13.1	-12.4	-12.5	-12.6	-12.9	24.2
24.3	25.2	24.2	24.8	24.7	25.0	-12.0	24.5
25.2	24.8	24.6	25.1	24.9	25.0	-12.1	24.2
24.7	24.8	-11.8	-13.1	-12.7	-12.2	-12.3	25.1
24.5	24.7	24.6	24.5	24.5	24.7	25.2	25.0

25.1	24.4	-15.1	-14.8	-16.0	-16.6	-15.9	24.3
24.9	24.9	25.1	24.5	-15.7	24.1	24.2	24.3
24.4	24.2	24.5	24.4	-15.3	24.2	24.9	24.2
24.3	24.5	24.7	24.6	-14.8	24.4	24.6	24.9
25.2	25.2	24.8	25.0	-15.4	24.3	24.9	25.0
24.1	24.2	-16.0	24.6	-15.0	24.3	24.3	24.3
24.7	25.2	-15.3	-15.7	-15.5	24.2	24.9	24.2
24.9	25.2	25.1	25.1	24.1	24.4	24.2	24.1

24.5	24.6	-10.7	-10.4	-10.2	-10.4	-11.1	24.0
24.6	23.6	23.8	24.3	-12.2	24.5	24.1	24.5
24.2	24.3	24.1	24.0	-12.1	24.4	24.2	23.9
24.3	24.1	24.3	23.7	-12.1	24.5	24.6	24.6
24.2	23.9	24.5	23.9	-11.7	23.7	23.9	24.4
23.9	24.4	23.7	23.8	-10.6	24.4	24.1	23.8
24.3	23.9	24.4	24.2	-10.9	24.0	23.8	23.8
23.8	23.6	24.2	24.5	23.7	24.6	24.6	24.1

24.3	24.3	-15.2	24.6	23.9	24.2	-15.2	24.3
24.0	24.3	-14.1	24.5	24.3	24.2	-13.7	24.4
23.8	24.6	-14.8	24.2	23.9	24.6	-14.3	24.3
23.9	24.4	-15.3	24.1	24.2	24.6	-14.5	24.5
23.8	24.2	-14.9	23.9	24.2	24.2	-14.0	24.3
24.1	23.7	-15.3	23.8	24.1	23.8	-15.6	23.7
24.5	23.9	-15.1	-14.4	-13.8	-14.4	-13.8	23.9
24.5	23.8	24.2	23.8	23.8	24.3	24.6	23.9

Fig. S22. Static spatially resolved thermal mapping. (**A**) Schematic representations of localized patterns shaped as letters “S”, “J”, “T”, and “U” generated on an 8×8 temperature sensor array. (**B**) Thermal distribution maps based on real-time sensor outputs, highlighting the array’s high spatial resolution. (**C**) Corresponding quantitative temperature matrices obtained from the sensor array, revealing localized regions with temperatures as low as -18 °C.

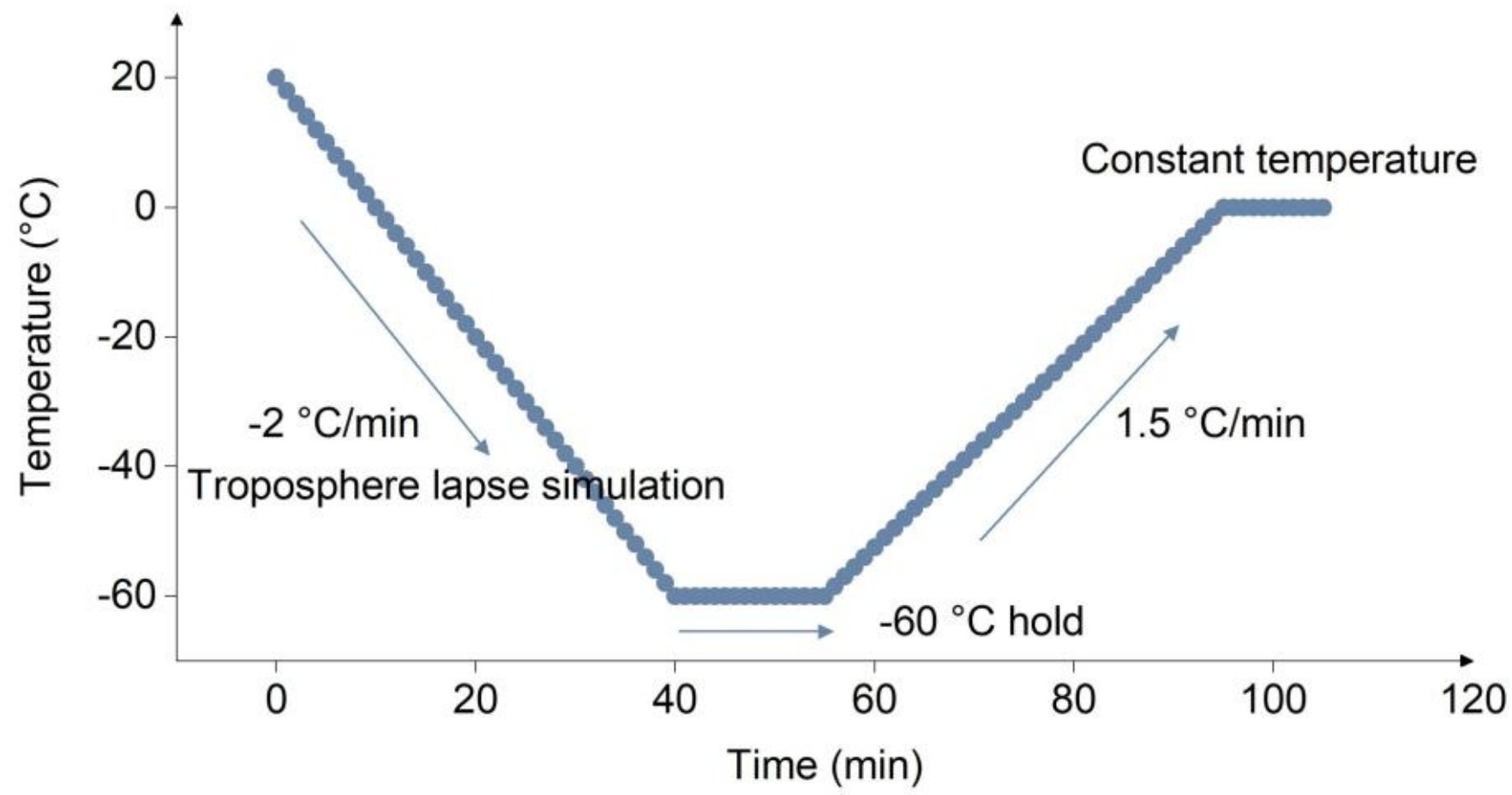

Fig. S23. Balloon ascent temperature trajectory used for low-temperature sensing evaluation. Programmed thermal profile mimicking atmospheric temperature variation during meteorological balloon ascent, including cooling from ~20 °C to -60 °C followed by gradual warming toward 0 °C.

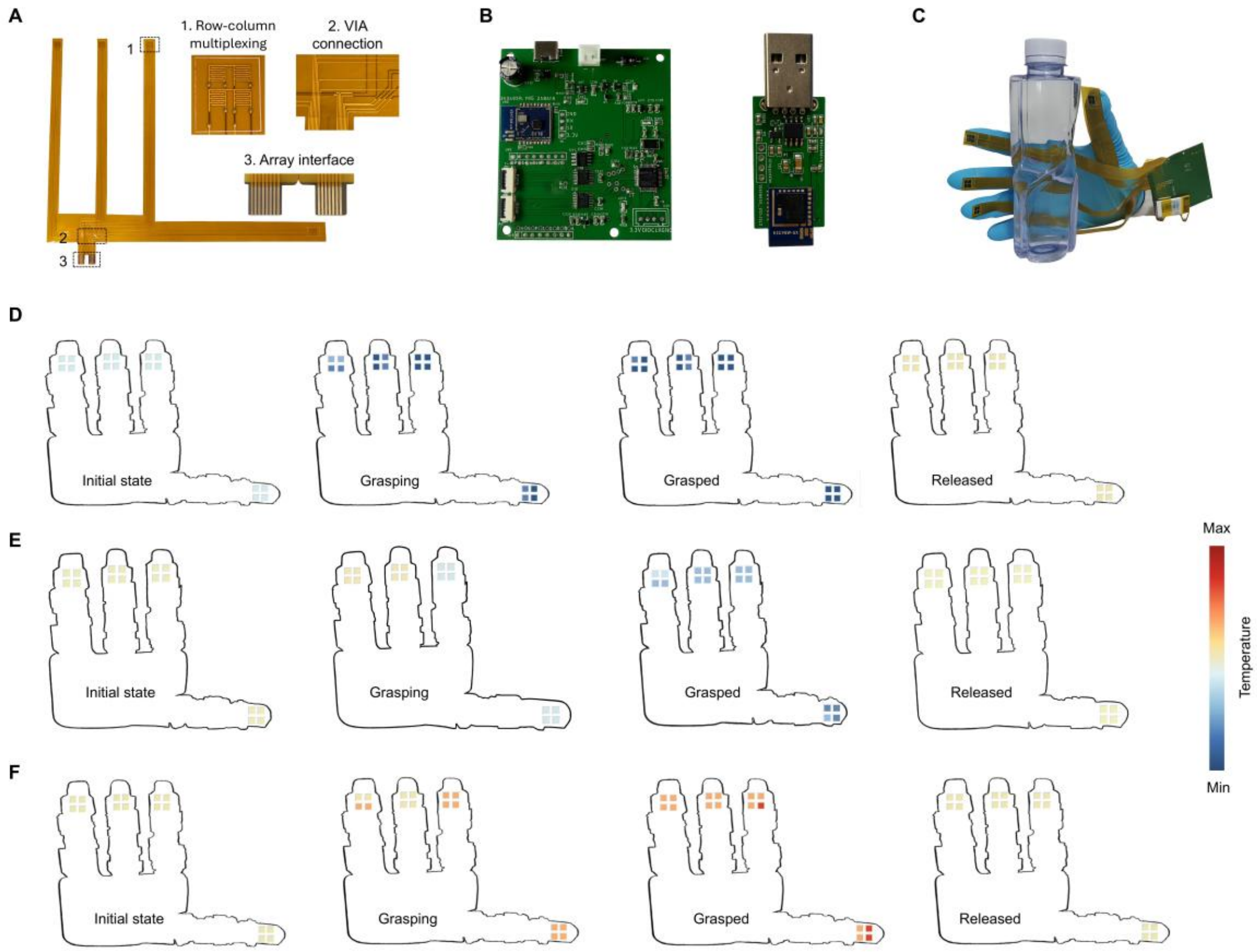

Fig. S24. Integrated hardware platform and real-time temperature mapping. (**A**) Design of the flexible sensing interface used for the 16-channel temperature array, including the row-column multiplexing architecture, vertical interconnect access (VIA) connections, and array interface for external circuitry. (**B**) Custom signal-acquisition electronics for array readout, consisting of the main control board and a compact USB data-acquisition module enabling communication with a host computer through LabVIEW. (**C**) Demonstration of the flexible sensor array integrated onto human hand for temperature perception. (**D-F**) Real-time temperature mapping of the human hand during object grasping under different thermal conditions. Sequential states include the initial state, grasping, grasped, and released stages. The color scale represents the measured temperature distribution across the 16 sensing channels, ranging from low (blue) to high (red). The maps correspond to interactions with objects at different temperatures: (**D**) cold object, (**E**) ambient temperature object (0-10°C), and (**F**) hot object (above 40°C). Data were acquired and visualized in real time using a LabVIEW-based acquisition system.

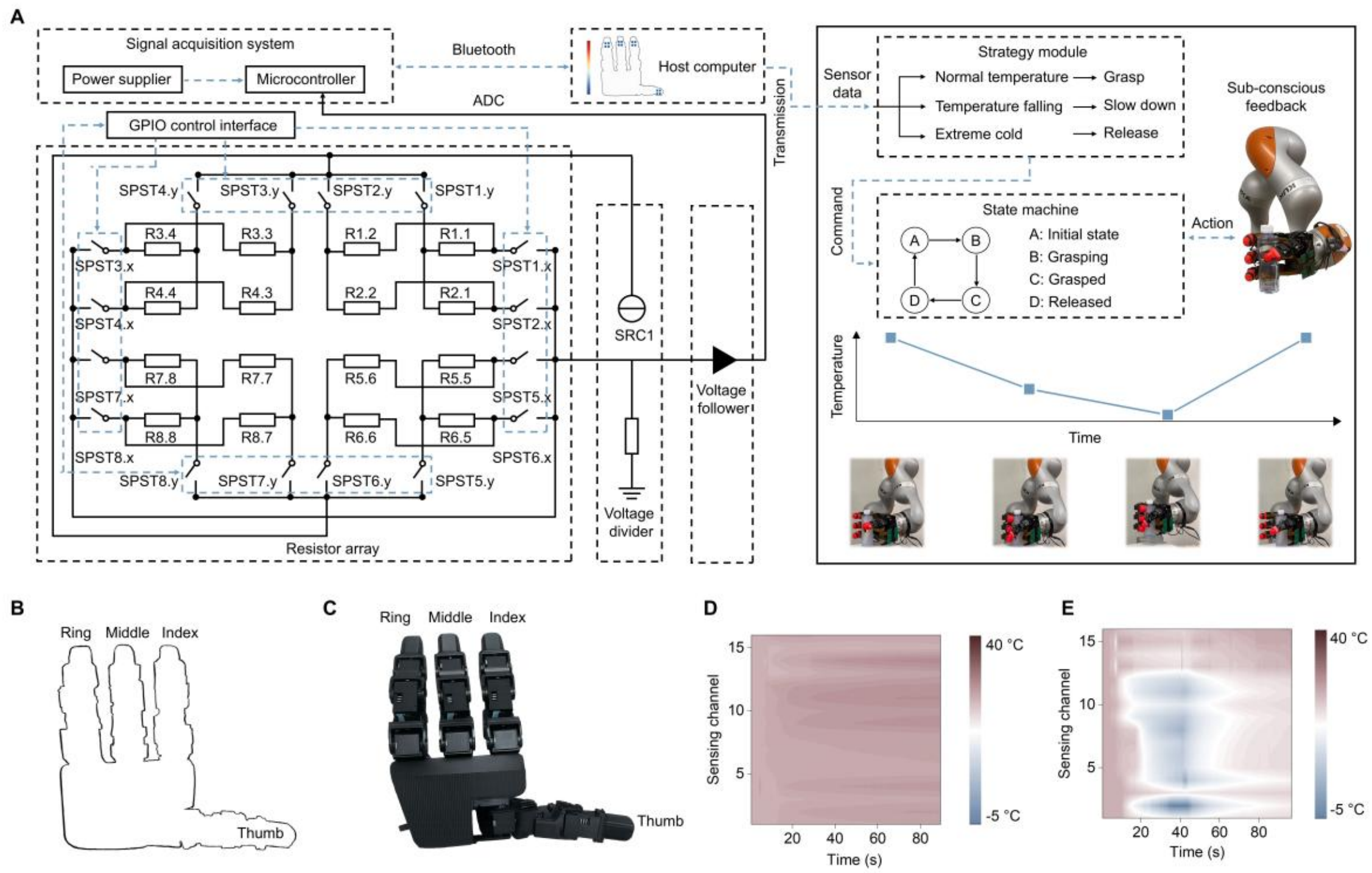

Fig. S25. System architecture and robotic hand integration for temperature perception. (**A**) Schematic illustration of the signal acquisition and control system for the temperature sensing array. The circuit consists of a resistor array addressed through row-column multiplexing using GPIO-controlled switches, followed by voltage division, buffering, and analog-to-digital conversion for data transmission to the host computer via Bluetooth. The acquired temperature signals are processed by a strategy module that determines grasping behavior based on temperature variations, enabling adaptive responses through a state-machine framework (initial state, grasping, grasped, and released). (**B**) Outline of the robotic hand, including the thumb, index, middle, and ring fingers, used as the platform for sensor integration. (**C**) Photograph of the four-finger robotic hand prototype used for thermal sensing and closed-loop feedback demonstration. (**D** and **E**) Representative spatiotemporal temperature maps recorded from the sensing array during robotic manipulation. The color scale indicates the measured temperature distribution across the sensing channels as a function of time, illustrating stable temperature perception during interaction with objects under different thermal conditions (normal and cold conditions).

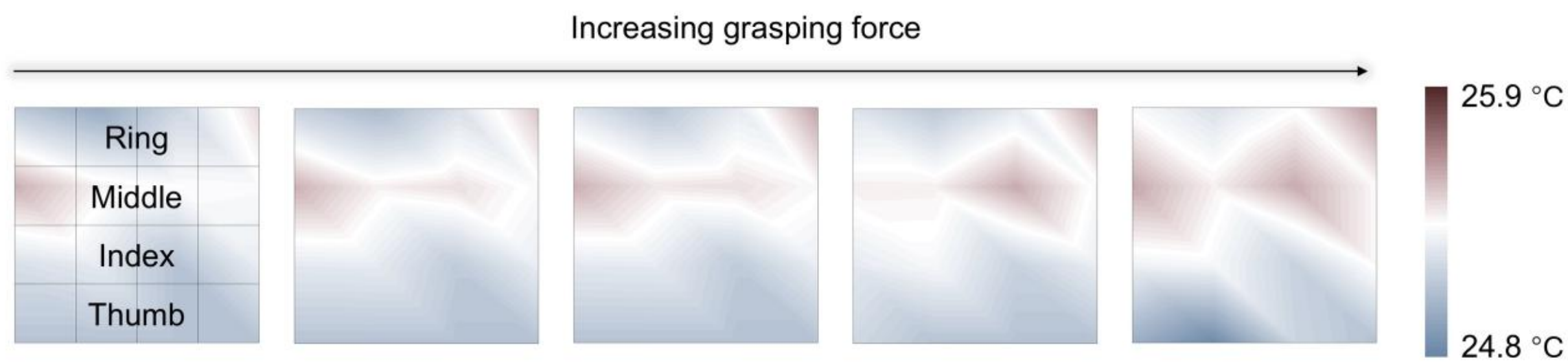

Fig. S26. Thermal mapping of a robotic hand grasping a room-temperature object under increasing force. Thermal maps recorded during robotic grasping with progressively increasing mechanical force. The temperature distribution remains stable within the room-temperature range (24.8-25.9 °C) during force variation.

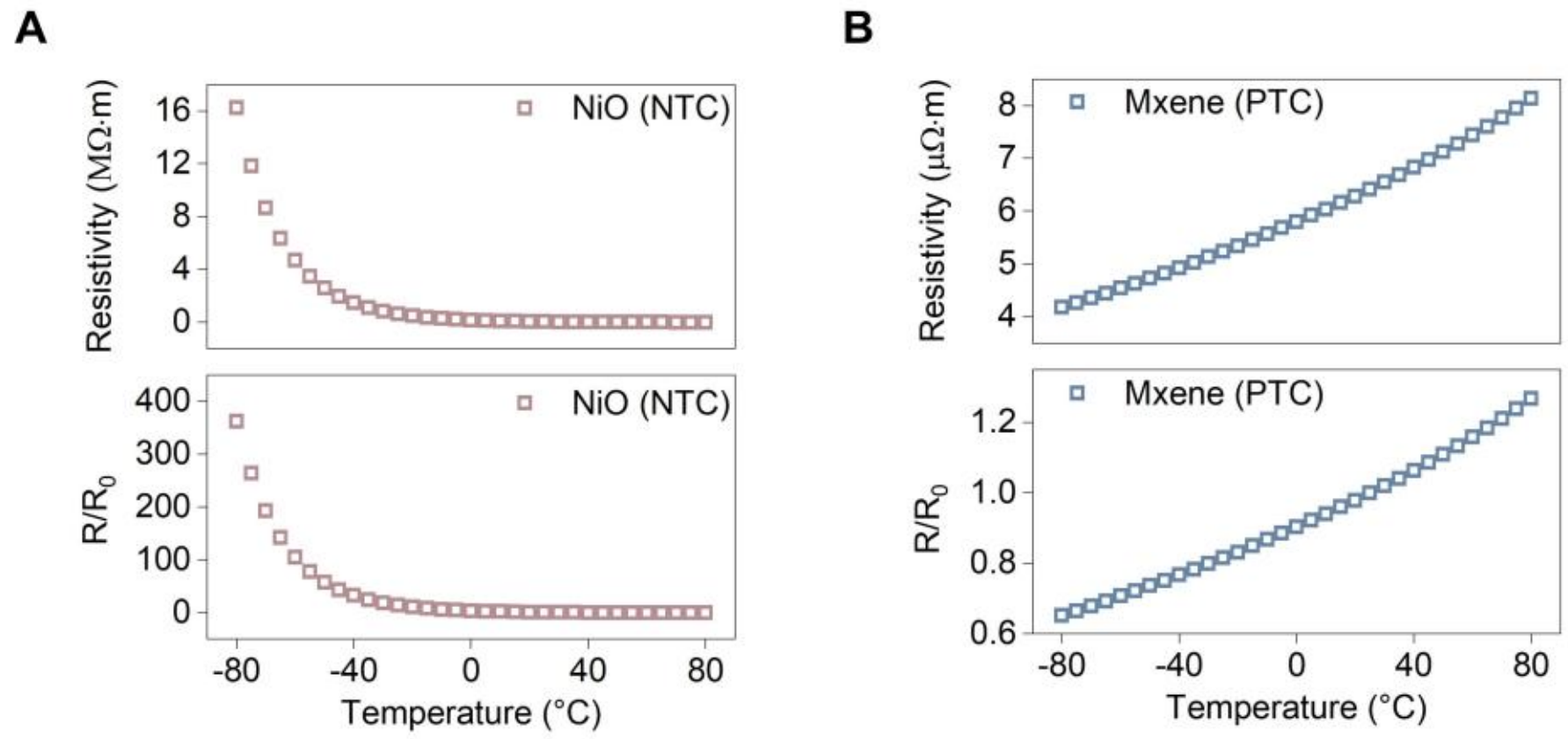

Fig. S27. Temperature-dependent electrical characteristics of individual conductive components. (**A**) Temperature-dependent resistivity and normalized resistance (R/R_0) of NiO, showing NTC behavior. (**B**) Temperature-dependent resistivity and normalized resistance (R/R_0) of MXene, showing PTC behavior.

A 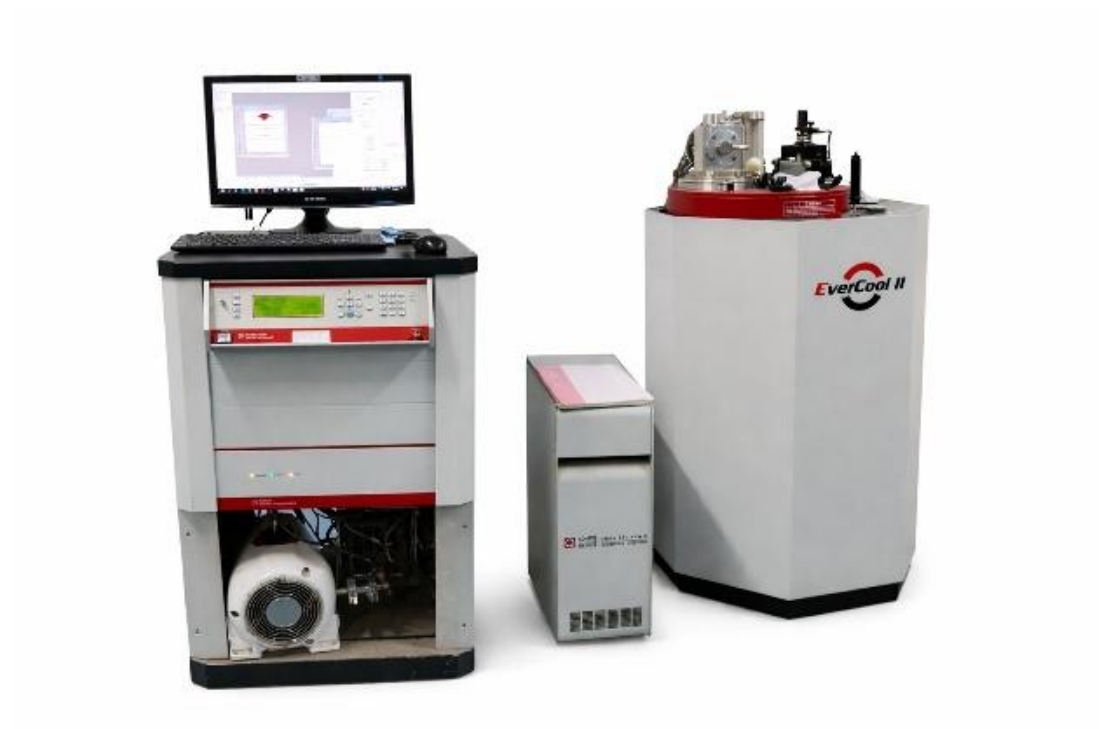

B 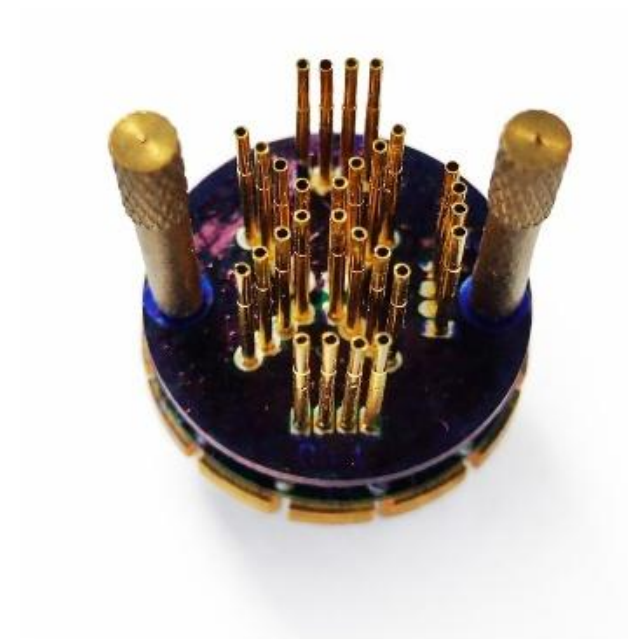

Fig. S28. Experimental setup for high-resolution temperature sensing measurements, which is performed by a third-party testing institution. (**A**) Quantum Design PPMS-9 platform used for precise temperature control during the sensing characterization, providing temperature stability down to 0.01 °C. (**B**) Electrical connection interface used for low-noise measurements of the sensing element.

Table S1. Comparison of reported temperature sensors using either electronic or ionic conduction mechanisms.

Type	Materials	Sensing range (℃)	│TCR_{max}│ (% ℃$^{-1}$)	Year	References
Electronic mode	NiO NP	25 to 70	9.2 (25 to 30 ℃)	2020	(14)
	PU/PVA/MXene	0 to 80	5.27 (0 to 30 ℃)<br>1.11 (30 to 80 ℃)	2022	(12)
	Ag_2S	25 to 85	4.7 (77.5 to 80 ℃)	2022	(13)
	MoS_2	30 to 70	1 (30 to 70 ℃)	2023	(16)
	NiO/CNTF	-15 to 60	20.2 (-15 to -10 ℃)<br>9.1 (-15 to 25 ℃)<br>2.1 (25 to 60 ℃)	2024	(15)
	rGO/MnO_2/TPU/ Kevlar	25 to 50	1.4 (25 to 60 ℃)	2024	(18)
Ionic mode	Poly (ionic liquid)	-20 to 60	11.3 (-20 to 25 ℃)<br>2.1 (25 to 60 ℃)	2020	(38)
	Deep eutectic solvents	30 to 90	8.42 (at 27 ℃)	2023	(25)
Supramolecular system	TPU/CMC/MXen e/NiO	-80 to 80	22, 280 (-80 to -75 ℃)	2026	This work

Movie S1. Stable grasping of a room-temperature object without reflexive response.

Movie S2. Reflexive release of the robotic hand triggered by contact with a cold object.
